# PSMD1 inhibition suppresses tumor progression and enhances antitumor immunity by modulating the RTKN/β-catenin/PD-L1 axis in hepatocellular carcinoma

**DOI:** 10.1038/s41419-025-08241-4

**Published:** 2026-01-14

**Authors:** Xiangjun Qian, Kai Zhang, Chao Ma, Yang Ji, Xianzhou Zhang, Li Wang, Tao He, Haibo Yu, Hao Zhuang, Xiaopei Hao

**Affiliations:** 1 Department of Hepatobiliopancreatic Surgery, The Affiliated Cancer Hospital of Zhengzhou University & Henan Cancer Hospital, Zhengzhou, China; 2 Hepatobiliary Center, Key Laboratory of Liver Transplantation, NHC Key Laboratory of Living Donor Liver Transplantation, The First Affiliated Hospital of Nanjing Medical University, Chinese Academy of Medical Sciences, Nanjing, China; 3 Department of Hepatobiliary Surgery, People’s Hospital of Zhengzhou University, Zhengzhou, China

**Keywords:** Cancer immunotherapy, Tumour immunology

## Abstract

Immunotherapy has emerged as a promising approach in the management of cancer. However, the suboptimal efficacy of immunotherapy monotherapy underscores the need to develop more effective combination strategies. In this study, we focused on PSMD1 to investigate its role and the molecular pathways by which it regulates the response to immunotherapy in hepatocellular carcinoma (HCC). In HCC, elevated PSMD1 levels are linked to associated with poor prognosis. PSMD1 was predominantly expressed in malignant epithelial cells. Tissue microarray results showed that PSMD1 was highly expressed in tumor tissues. Silencing PSMD1 suppressed HCC cell proliferation and promoted apoptosis in both in vitro and in vivo models. Additionally, PSMD1 suppression decreased PD-L1 expression, thereby enhancing the therapeutic efficacy of anti-PD-1 therapy. Mechanistically, publicly available single-cell RNA sequencing (scRNA-seq) datasets indicated that PSMD1 positively regulates β-catenin signaling. Silencing of PSMD1 decreased the expression of β-catenin pathway-associated proteins. Further analysis via mass spectrometry revealed that PSMD1 interacts with Rhotekin (RTKN) and suppresses its ubiquitination. Subsequent experiments revealed that RTKN enhances β-catenin expression through AKT phosphorylation, thereby increasing PD-L1 transcription. In summary, our findings demonstrate that PSMD1 regulates RTKN protein expression, whereas RTKN facilitates β-catenin expression via AKT phosphorylation. This mechanism contributes to HCC progression and the effectiveness of immunotherapy. The PSMD1/RTKN/β-catenin axis could serve as a promising therapeutic target for HCC.

## Introduction

Hepatocellular carcinoma (HCC), the most common type of primary liver cancer, is the sixth most commonly diagnosed malignancy worldwide and the third leading cause of cancer-related mortality globally [1]. The asymptomatic presentation of early-stage HCC often leads to delayed diagnosis, rendering many patients ineligible for curative surgical intervention. At present, multikinase inhibitors and immune checkpoint inhibitors (ICIs) constitute the cornerstone of therapeutic strategies for advanced HCC [2]. Among these agents, anti-PD-1 and anti-PD-L1 monoclonal antibodies have transformed the therapeutic landscape of solid tumors, including HCC [3]. Although a substantial number of patients have benefited from immunotherapy, including those with advanced HCC who have subsequently become eligible for surgical resection, the efficacy of immune checkpoint blockade (ICB) remains suboptimal, with response rates averaging only 20% [4]. Even when combined with targeted therapies, the overall response rate does not surpass 40% [5], highlighting the urgent need for more effective therapeutic strategies to address the unmet needs of a significant patient population. Consequently, innovative strategies to increase the clinical efficacy of ICB in HCC are urgently needed.

With the advancement of detection technologies, the diversity of detection methods leads to increased variability in the generated data. To integrate these data and identify underexplored therapeutic targets, we utilized machine learning to train data models and identify genes associated with the efficacy of immunotherapy [6]. PSMD1 (proteasome 26S subunit, non-ATPases 1), as the most prominent gene, was selected as the focus of our study. Proteasomes are multisubunit proteases that mediate the degradation of ubiquitinated proteins. PSMD1, a member of the 19S regulatory complex in the 26S proteasome, has been implicated in the progression of multiple malignancies through diverse mechanisms. For example, it promotes p53 degradation in breast cancer [7], correlates with poor prognosis in oropharyngeal cancer [8], and inhibits tumor cell apoptosis in lung adenocarcinoma [9]. In chronic myeloid leukemia, PSMD1 stabilizes P65 by inhibiting its ubiquitination, thereby driving disease progression [10]. While posttranslational modifications of PSMD1 have been shown to modulate its effects on ubiquitination [11], the role of PSMD1 in HCC progression and its impact on immunotherapy remain unexplored.

In this study, through bioinformatics analysis, PSMD1 was identified as highly expressed in HCC and inversely correlated with immunotherapy efficacy. Subsequent knockdown experiments revealed that PSMD1 suppresses HCC proliferation, induces apoptosis, and enhances sensitivity to PD-1 blockade. Mechanistically, mass spectrometry (MS) analysis demonstrated that PSMD1 interacts with Rhotekin (RTKN), stabilizing its protein levels by inhibiting ubiquitination. Further experiments confirmed that RTKN activates the β-catenin signaling pathway via AKT-mediated GSK3β phosphorylation, ultimately increasing PD-L1 transcription. This study provides novel insights into the mechanism through which PSMD1 modulates the β-catenin pathway, promotes PD-L1 expression and influences the immunotherapy response. These findings highlight the potential of PSMD1 as both a prognostic biomarker and a therapeutic target to increase the efficacy of ICB therapy in HCC.

## Results

### PSMD1 is an oncogene associated with HCC progression and clinical outcomes

**Fig. 1:**
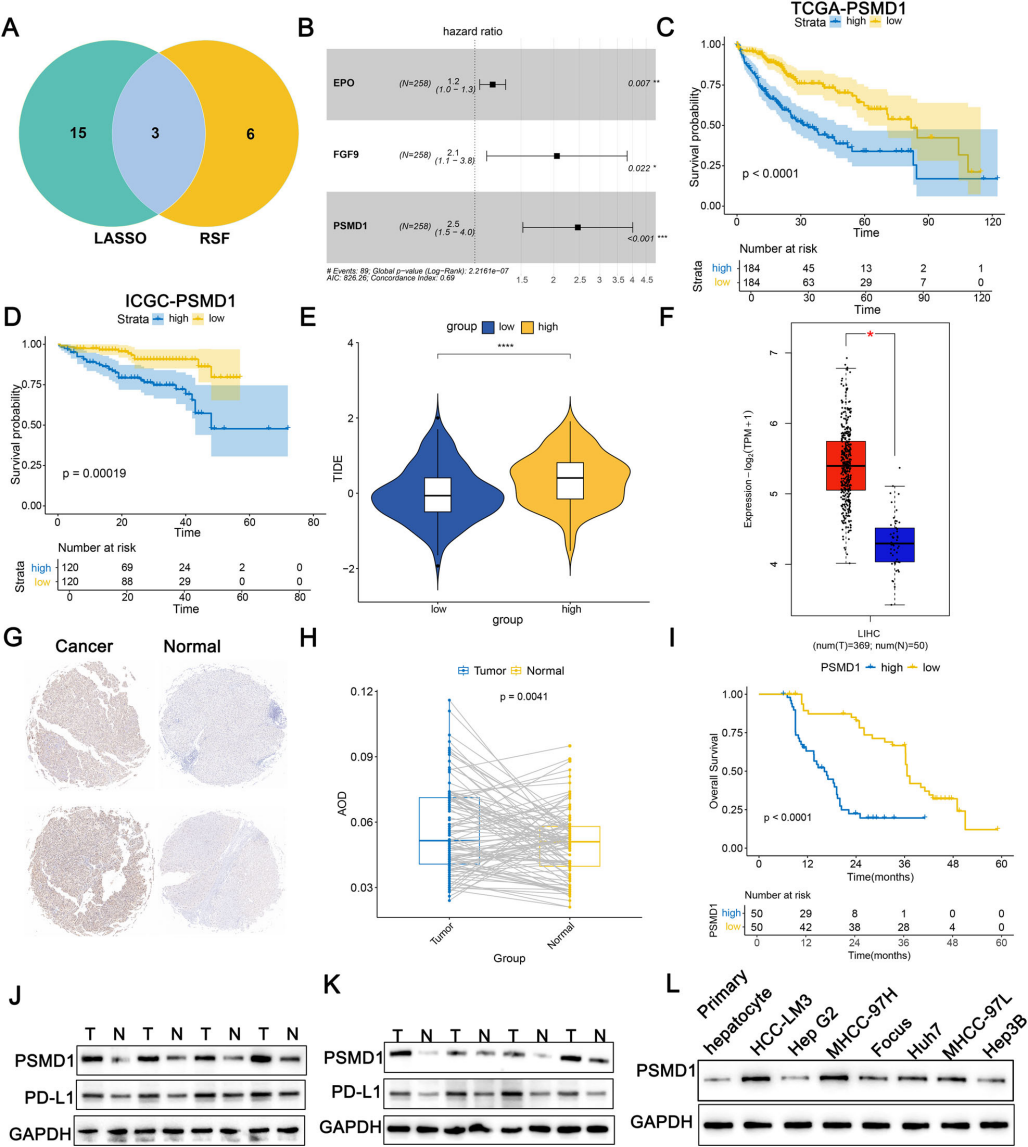
PSMD1 is an oncogene associated with HCC progression and clinical outcomes. **A** Venn diagram showing overlapping genes identified by the LASSO and RSF methods. **B** A forest plot of OS was generated via multivariate Cox regression models. Kaplan–Meier curves of OS according to the risk score in TCGA-LIHC (**C**) and ICGC_LIRI_JP (**D**) cohorts. **E** Tumor immune dysfunction and exclusion (TIDE) scores for the prediction of immunotherapy response in the two groups. **F** Boxplot showing significant differences in PSMD1 mRNA levels between tumor and normal samples in the TCGA-LIHC cohort. **G**, **H** Immunohistochemistry (IHC) analysis of PSMD1 protein levels in paired tumor and adjacent normal tissues. **I** The Kaplan–Meier survival curve for PSMD1 expression revealed that patients in the high-PSMD1 expression group had worse OS. **J**, **K** Protein expression levels of PSMD1 and PD-L1 in HCC tissues and corresponding adjacent tissues. **L** Protein expression levels of PSMD1 and PD-L1 in primary human hepatocytes and HCC cell lines. *p < 0.05; **p < 0.01; ***p < 0.001. The data are shown as the means ± SEMs.

To explore the genetic factors underlying HCC progression and their influence on immune and targeted therapies, the TCGA dataset comprising 368 HCC cases was partitioned into two cohorts: 258 cases for training and 110 cases for validation, maintaining a 7:3 ratio. A univariate Cox regression analysis conducted on the training cohort identified 306 genes with prognostic significance. Subsequent LASSO regression narrowed this down to 18 pivotal genes (Fig. S1A, B). Additionally, random survival forest (RSF) analysis identified nine key genes (Fig. S1C). The overlap between these two methods highlighted three genes subjected to multivariate Cox regression analysis (Fig. 1A). Among these, PSMD1 presented the highest hazard ratio (HR = 2.50) (Fig. 1B). The cohorts were then stratified into high- and low-risk groups on the basis of their median risk scores. Survival analyses demonstrated that individuals in the high-risk group exhibited markedly reduced overall survival compared with their low-risk counterparts (Fig. 1C, D; Fig. S1D, E). The ESTIMATE algorithm revealed a greater degree of immune infiltration in the high-risk group (Fig. S1F–H). CIBERSORT analysis revealed an increased abundance of regulatory T cells and M0 macrophages in this group. In addition, TIDE analysis confirmed that the high-risk group exhibited elevated TIDE scores, indicating a poorer immunotherapeutic response (Fig. 1E). To further investigate the role of PSMD1 in HCC progression and its relationship with immunotherapy, a comprehensive analysis was conducted. Data from the TCGA database revealed significant overexpression of PSMD1 in HCC tissues (Fig. 1F). Immunohistochemical analysis of tissue microarrays from 100 patients, comprising both cancerous and adjacent noncancerous tissues, revealed elevated PSMD1 levels in HCC tissues (Fig. 1G, H). Patients exhibiting increased PSMD1 expression experienced notably reduced overall survival (Fig. 1I). Western blot validation confirmed the increased PSMD1 levels in the HCC samples (Fig. 1J, K). Previous studies have suggested that elevated PD-L1 expression on the cell surface can transform Th1 cells expressing T-box transcription factors into FOXP3+T regulatory cells in vivo, thereby amplifying immunosuppressive effects [12]. By integrating TIDE and CIBERSORT data, this study evaluated the expression patterns of PSMD1 and PD-L1 and revealed a concurrent increase in PD-L1 levels, which was consistent with PSMD1 upregulation in HCC tissues (Fig. 1J, K, S1J). Overall, PSMD1 overexpression in HCC tissues contrasted with its reduced expression in adjacent noncancer tissues. These findings prompted further analysis of PSMD1 expression in relation to the clinicopathological characteristics of 100 HCC patients. Higher PSMD1 expression levels were associated with larger tumor size (p = 0.005), advanced TNM stage (p = 0.015), and increased Edmondson grade (p = 0.005) (Table 1). As a biomarker highly expressed in HCC, PSMD1 holds promise for guiding therapeutic strategies and improving prognostic assessments in HCC patients.

**Table 1:** Correlation between PSMD1 expression and clinicopathological features in HCC tissues (n = 100, χ2-test).

	PSMD1 expression			
Variables		high	low	*P*-value
		50	50	
Age (year)				0.317
<60		23	29	
≥60		27	21	
Gender				0.402
Male		35	30	
Female		15	20	
Liver cirrhosis				0.222
Yes		26	33	
No		24	17	
AFP (ng/mL)				0.109
≤200		31	28	
>200		19	22	
HBsAg				0.435
positive		39	43	
negative		11	7	
Tumor size				0.005*
<5 cm		8	22	
≥5 cm		42	28	
TNM stage				0.015*
I–II		15	28	
III–IV		35	22	
Edmondson grade				0.005*
I–II		17	32	
III–IV		33	18	

*P < 0.05.

### Identification of the cellular localization and cell communication pathways associated with PSMD1

**Fig. 2:**
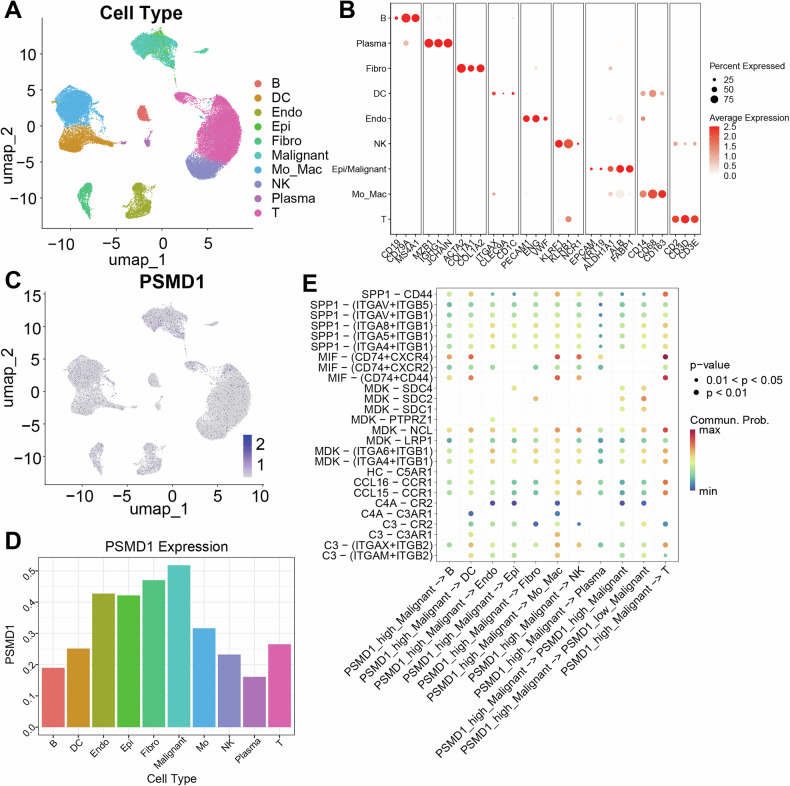
Identification of the cellular localization and cell communication pathways associated with PSMD1. **A** UMAP plots showing the identification of different clusters of cells in human HCC tumors. **B** Dot plots showing the specific cell markers for different cell types. UMAP plot (**C**) and bar plot (**D**) indicating PSMD1 expression across different cell clusters. **E** CellChat analysis revealed the probabilities of communication from high- and low-PSMD malignant cells to other cell clusters. *p < 0.05; **p < 0.01; ***p < 0.001. The data are shown as the means ± SEMs.

To further investigate the distribution and function of PSMD1 within the TME, we conducted a single-cell analysis. After performing quality control and filtering on the GSE149614 dataset, 49,431 cells were retained for subsequent analyses. Using classical cell markers, the cells were categorized into various types, including B cells, dendritic cells, endothelial cells, epithelial cells, fibroblasts, malignant tumor cells, monocytes/macrophages, NK cells, plasma cells, and T cells (Fig. 2A, B). PSMD1 expression was detected across all cell types, with the highest levels observed in HCC tumor cells (Fig. 2C, D). To explore the potential impact of PSMD1 expression in tumor cells on the tumor microenvironment, we conducted a cell communication analysis. We defined tumor cells exhibiting the top 10% of PSMD1 expression as PSMD1-high malignant cells, while the remaining tumor cells were classified as PSMD1-low malignant cells. Subsequent analysis utilizing the CellPhoneDB-secreted ligand‒receptor database revealed that tumor cells with high PSMD1 expression predominantly employ ligands such as MIF, MDK, and SPP1 as signaling molecules to communicate with immune cells within the microenvironment (Fig. 2E). In conclusion, PSMD1 plays an important role in the HCC microenvironment.

### Inhibition of PSMD1 inhibits HCC proliferation and accelerates apoptosis in vitro

**Fig. 3:**
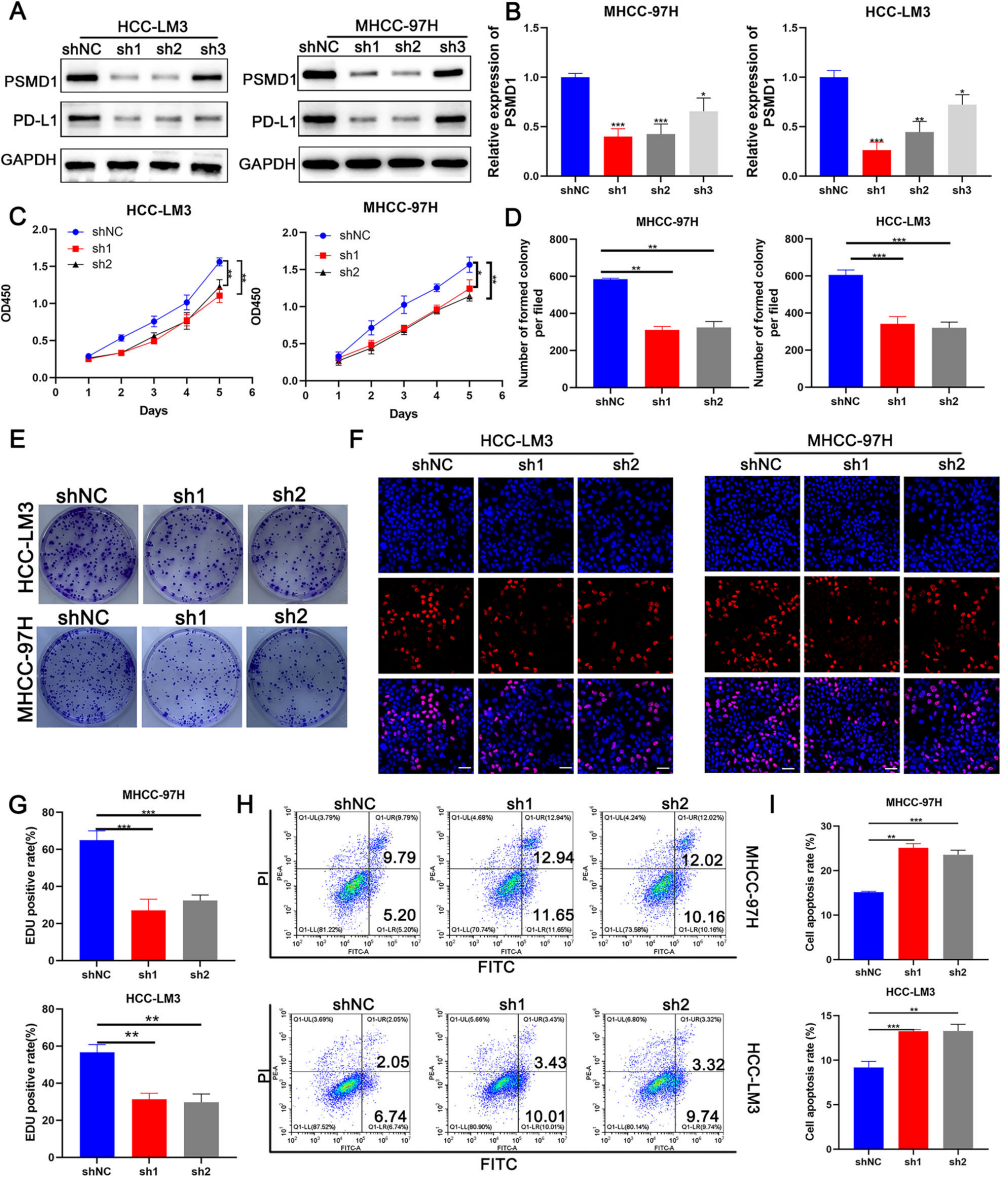
PSMD1 inhibition inhibits HCC proliferation and accelerates apoptosis in vitro. **A** Western blotting of PSMD1 and PD-L1 expression in HCC-LM3 and MHCC-97H cells transfected with shPSMD1. **B** MRNA expression of PSMD1 in HCC-LM3 and MHCC-97H cells transfected with shPSMD1. **C** A CCK-8 assay was used to determine the growth rate of PSMD1-knockdown HCC cells. **D**, **E** Colony formation assay for HCC cells. **F**, **G** An EdU assay was performed to evaluate the proliferation ability of HCC cells after PSMD1 was downregulated. Scale bar, 50 μm. **H**, **I** Cell apoptosis assays were performed in the PSMD1-knockdown groups. *p < 0.05; **p < 0.01; ***p < 0.001. The data are shown as the means ± SEMs.

To investigate the role of PSMD1 in HCC progression, we conducted knockdown experiments in which PSMD1 was targeted in MHCC-97H and HCC-LM3 cell lines via shRNAs. Two specific shRNA sequences, referred to as sh1 and sh2, were employed to generate shPSMD1-1 and shPSMD1-2 constructs, respectively (Fig. 3A, B). The results from the CCK8 assays demonstrated that PSMD1 silencing resulted in a significant reduction in cell proliferation (Fig. 3C). Similarly, colony formation and EdU assays revealed a markedly lower average colony count in PSMD1-suppressed cells than in control cells (Fig. 3D, E). Furthermore, apoptosis assays revealed an increased apoptotic rate in HCC cells treated with PSMD1 shRNA (Fig. 3H, I). To further investigate apoptosis-associated proteins, their expression levels were analyzed via western blotting. BCL-2 expression was significantly lower in PSMD1-knockdown cells from both the HCC-LM3 and MHCC-97H lines than in control cells. Conversely, the expression of BAX was notably upregulated in these knockdown cells (Fig. S1K). Taken together, these results indicate that PSMD1 knockdown inhibits HCC cell proliferation while promoting apoptosis in vitro.

### PSMD1 regulates the expression of PD-L1 through the β-catenin signaling pathway

**Fig. 4:**
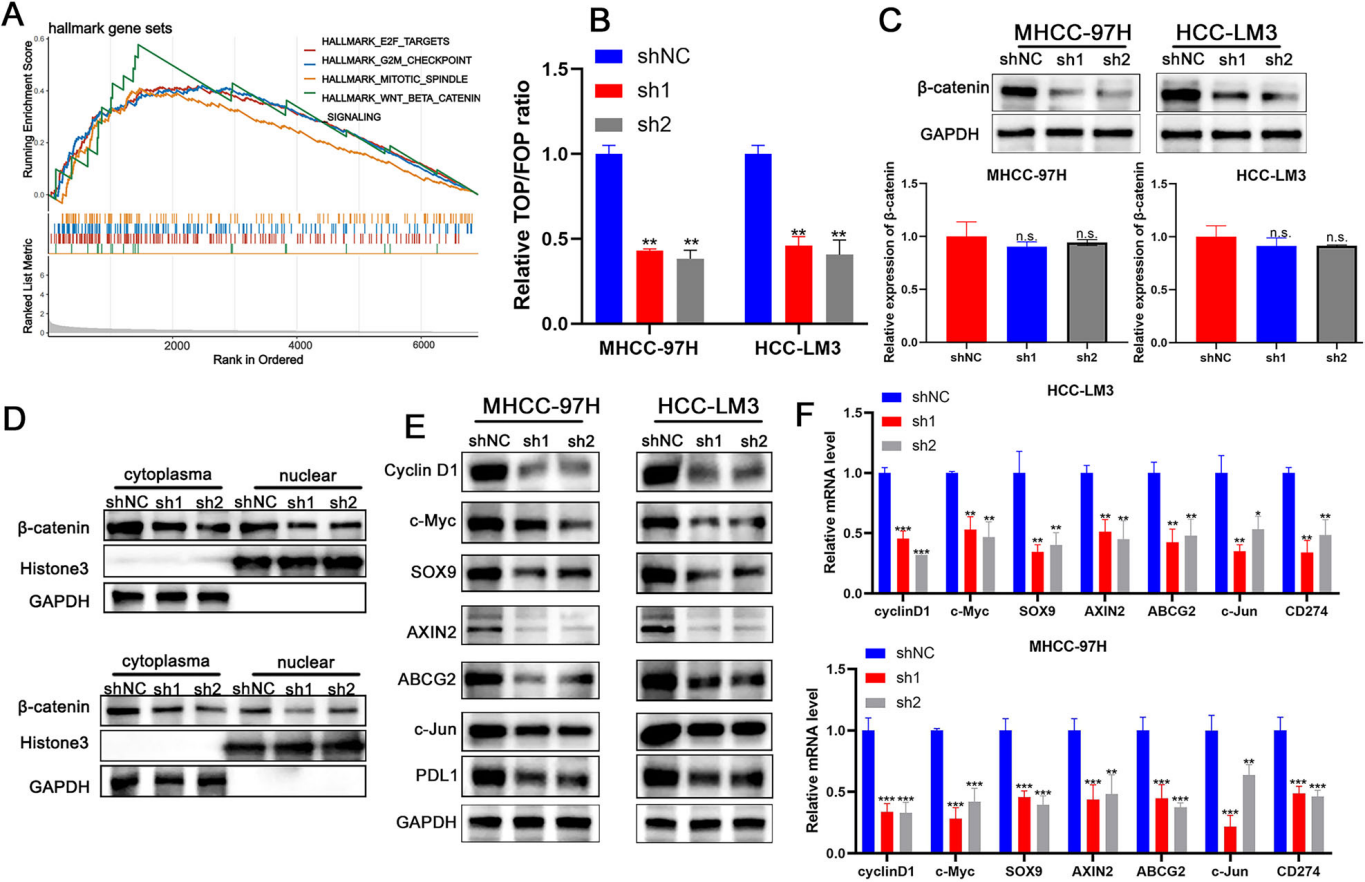
PSMD1 regulates the expression of PD-L1 through the Wnt/β-catenin signaling pathway. **A** GSEA result showing the pathways enriched with PSMD1 expression in malignant cells. **B** The luciferase activity of β-catenin/Tcf4 transcriptional activity was measured in shPSMD1 and control HCC cells. **C** Protein and mRNA expression of β-catenin in HCC-LM3 and MHCC-97H cells transfected with shPSMD1. **D** Knockdown of PSMD1 decreased the protein levels of cytoplasmic and nuclear β-catenin in HCC cells. **E** Knockdown of PSMD1 decreased the protein levels of CyclinD1, c-Myc, SOX9, AXIN2, ABCG2, c-jun, PD-L1 in HCC cells. **F** Knockdown of PSMD1 decreased the mRNA levels of CyclinD1, c-Myc, SOX9, AXIN2, ABCG2, c-jun, and PD-L1 in HCC cells. *p < 0.05; **p < 0.01; ***p < 0.001. The data are shown as the means ± SEMs.

To elucidate the signaling pathways associated with the biological function of PSMD1, GSEA was performed using data from single-cell databases. The analysis revealed a correlation between elevated PSMD1 expression and increased activity of the β-catenin signaling pathway (Fig. 4A). Prior studies have established that the WNT/β-catenin/TCF signaling cascade plays a pivotal role in regulating the development and progression of HCC [2]. Consistent with these observations, the β-catenin/Tcf4 transcriptional reporter (TOP/FOPFLASH) luciferase assay revealed a significant reduction in β-catenin transcriptional activity in HCC cells following PSMD1 knockdown (Fig. 4B). Further investigation into the effect of PSMD1 on β-catenin revealed no measurable changes at the mRNA level. However, PSMD1 knockdown markedly reduced the total β-catenin protein level (Fig. 4C). Fractionation experiments further confirmed notable alterations in the β-catenin protein distribution between the nucleus and cytoplasm upon PSMD1 silencing (Fig. 4D). These changes in β-catenin signaling were accompanied by reduced expression of β-catenin target genes, including CyclinD1, c-Myc, SOX9, AXIN2, ABCG2, and c-jun, in both MHCC-97H and HCC-LM3 cells (Fig. 4E, F). Collectively, these findings provide strong evidence that PSMD1 significantly regulates β-catenin protein expression, ultimately impacting PD-L1 expression. Taken together, these findings suggest that the aberrant expression of the PSMD1/β-catenin axis contributes substantially to HCC progression.

### PSMD1 promotes tumor proliferation and inhibits apoptosis through the β-catenin pathway

On the basis of the hypothesis that β-catenin signaling operates downstream of PSMD1, a series of experiments was conducted to evaluate whether PSMD1 modulates β-catenin signaling during HCC progression. TOP/FOP reporter assays in HCC cells revealed that TOP/FOP luciferase activity was significantly reduced following sh1 transfection (Fig. S2A). However, this decrease in luciferase activity was reversed upon treatment with the Wnt/β-catenin agonist SKL2001 in MHCC-97H and HCC-LM3 cells (Fig. S2A). In addition, treatment of shPSMD1-transfected HCC cells with SKL2001 confirmed, via Western blot analysis, that β-catenin signaling was reactivated in the SKL2001-treated cells. This reactivation was accompanied by increased expression of β-catenin downstream targets, including Cyclin D1, c-Myc, SOX9, AXIN2, ABCG2, PDL1, and c-Jun (Fig. S2B). These findings suggest that PSMD1 knockdown significantly suppresses β-catenin signaling activity. In support of this hypothesis, SKL2001 effectively rescued the proliferative ability of HCC cells inhibited by PSMD1 knockdown, as demonstrated by CCK-8, colony formation, and EdU assays (Fig. S2C–G). Additionally, flow cytometry analysis demonstrated that SKL2001 treatment attenuated apoptosis in HCC cells with shPSMD1 knockdown (Fig. S3A, B).

### PSMD1 binds RTKN and inhibits its degradation in HCC cells

**Fig. 5:**
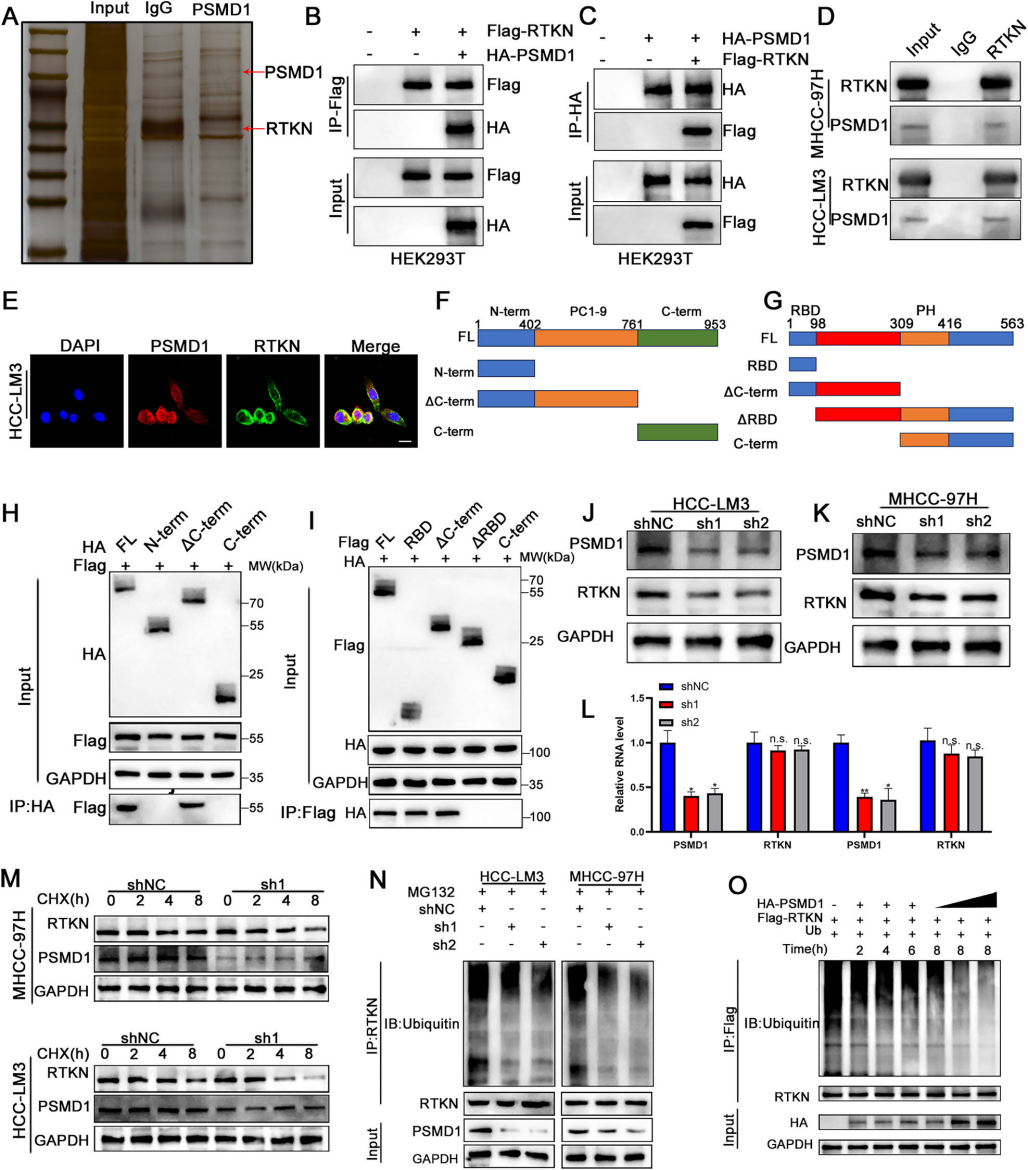
PSMD1 binds RTKN2 and inhibits its degradation in HCC cells. **A** Silver-stained SDS‒PAGE gel containing proteins derived from IP by PSMD1 and IgG. **B**, **C** A co-IP assay was performed in HEK293T cells. **D** A co-IP assay was performed in HCC cells. **E** Immunofluorescence analysis revealed the colocalization of RTKN (green) and PSMD1 (red) in HCC-LM3 cells. Scale bar, 20 μm**. F** Schematic drawing of full-length PSMD1 and truncations. **G** Schematic drawing of full-length RTKN and truncations. **H**, **I** Co-IP assays were performed in HEK293T cells transfected with Flag-RTKN and HA-PSMD1 truncations. **J**, **K** Knockdown of PSMD1 decreased the protein level of RTKN. **L** Knockdown of PSMD1 does not decrease the mRNA level of RTKN. **M** Western blot analysis of RTKN in MHCC-97H and HCC-LM3 cells treated with CHX. **N** Ubiquitination of RTKN proteins in HCC cells was determined via co-IP and western blotting. **O** PSMD1 inhibits the ubiquitination of RTKN in a time- and dose-dependent manner. *p < 0.05; **p < 0.01; ***p < 0.001. The data are shown as the means ± SEMs.

To investigate the specific mechanisms by which PSMD1 contributes to β-catenin, we performed coimmunoprecipitation (co-IP) followed by mass spectrometry (IP–MS) to identify potential targets of PSMD1 (Fig. 5A). Using IP‒MS analysis, we identified 116 distinct proteins (Table S1) and found that PSMD1 interacts with RTKN rather than directly binding to β-catenin (Fig. S3C). Notably, RTKN has been previously reported to increase β-catenin transcription [13]. To verify the interaction between these proteins, HEK293T cells were transfected individually or together with Flag-RTKN or HA-PSMD1, followed by detection with specific antibodies (Fig. 5B, C). Similarly, we confirmed that these proteins physically interact within HCC cells (Fig. 5D; Fig. S3D). Immunofluorescence staining revealed that PSMD1 (red) and RTKN (green) colocalized in the cytoplasm of HCC cells (Fig 5E and S3E). Additionally, to identify the minimal essential region necessary for their interaction, various RTKN and PSMD1 deletion mutants were co-expressed in HEK293T cells. As shown in Fig. 5F–I, the direct interaction is facilitated by the RBD domain of RTKN (amino acids 1--98) and the PC1--9 domain of PSMD1 (amino acids 402--761), which are necessary and sufficient. Interestingly, PSMD1, a component of the UPS (proteasome 26S subunit, non-ATPase 1), has been shown to inhibit the ubiquitination of target proteins [10]. Knockdown of PSMD1 dramatically reduced RTKN protein expression (Fig. 5J, K). In addition, the mRNA expression level of RTKN did not significantly change with PSMD1 knockdown (Fig. 5L). Similarly, after treatment with cycloheximide (CHX), PSMD1 knockdown facilitated the degradation of endogenous RTKN, suggesting that PSMD1 prolongs the half-life of the RTKN protein (Fig. 5M). We found that the knockdown of PSMD1 significantly reduced RTKN ubiquitination in HCC cells (Fig. 5N). In vitro ubiquitylation assays revealed that PSMD1 inhibited the ubiquitination of RTKN in a time- and dose-dependent manner (Fig. 5O). Taken together, these results indicate that PSMD1 is a regulator of RTKN and a prognostic marker.

### PSMD1 regulates the progression of HCC in an RTKN-dependent manner

Studies have reported that RTKN plays a role in promoting tumor progression in gastric cancer and HCC [14, 15]. Therefore, we hypothesize that PSMD1 may exert its biological functions through RTKN. To test this hypothesis, we further validated this observation using database analysis. Examination of RTKN expression in the TCGA database demonstrated significantly higher expression in HCC tissues compared to adjacent non-cancerous tissues. (Fig. S4A). Moreover, Kaplan-Meier survival analysis of the TCGA dataset revealed that elevated RTKN expression was associated with poorer overall survival (OS) in HCC patients, consistent with its elevated expression levels in tumor tissues (Fig. S4B). We next investigated whether PSMD1 regulates HCC progression through an RTKN-dependent mechanism. WB and RT-qPCR analyses confirmed the transfection efficiency of the RTKN plasmid, and we observed that RTKN does not affect the expression of PSMD1 (Fig. S3F, G). Consistent with our hypothesis, RTKN overexpression reversed the reduction in β-catenin caused by PSMD1 knockdown (Fig. S4C). Additionally, RTKN effectively rescued the suppression of HCC cell proliferation induced by PSMD1 knockdown, as evidenced by the results of the CCK-8, colony formation, EdU proliferation (Fig. S4D–H; Fig. S5A). Moreover, flow cytometry analysis revealed that RTKN overexpression significantly decreased apoptosis in PSMD1-knockdown HCC cells (Fig. S4I, J). Collectively, these findings suggest that RTKN overexpression modulates the tumor-inhibitory effects of PSMD1 knockdown in HCC.

### RTKN binds to AKT and promotes its phosphorylation

**Fig. 6:**
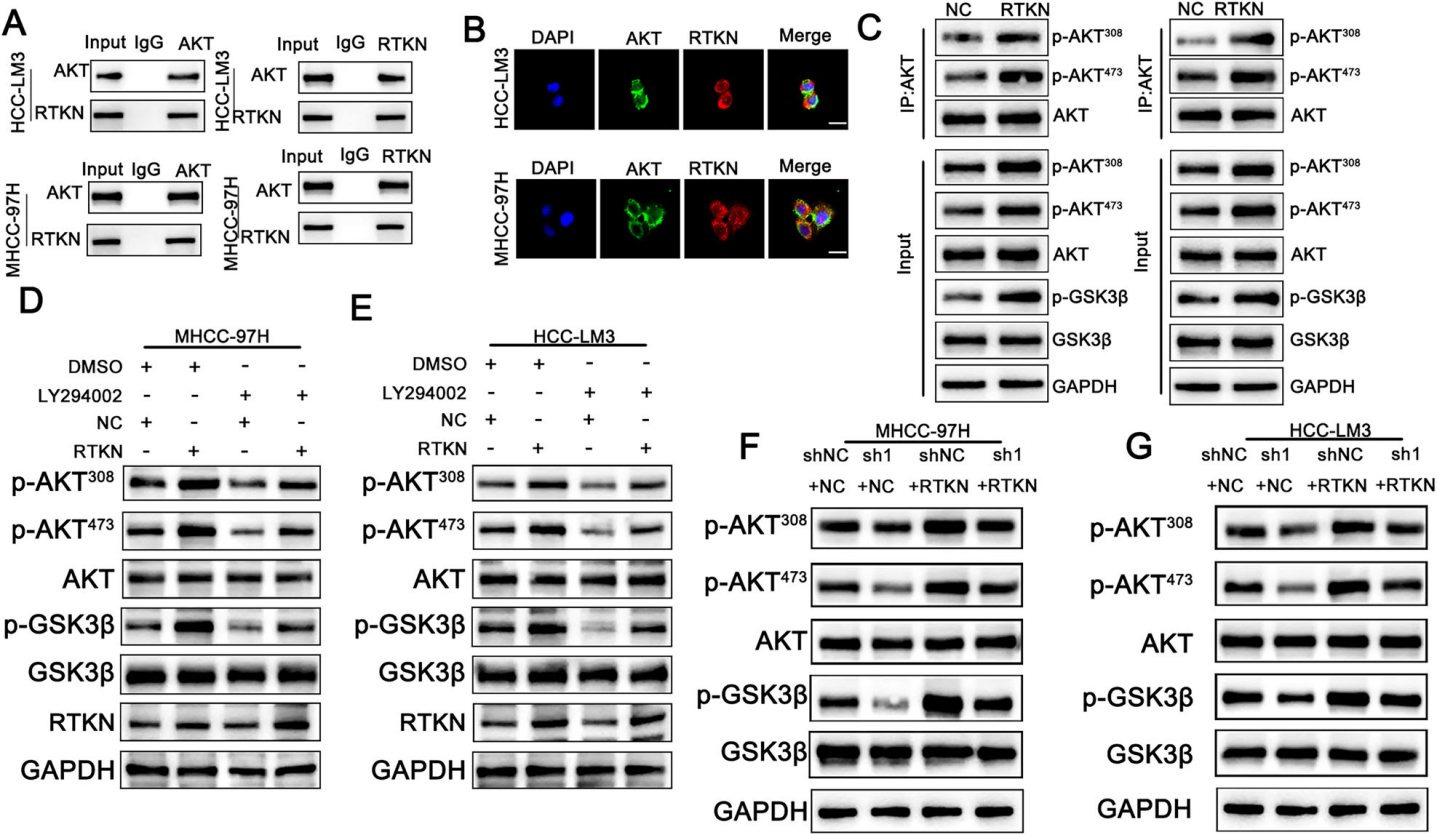
RTKN binds to AKT and promotes its phosphorylation. **A** A co-IP assay was performed in HCC cells. **B** Immunofluorescence analysis revealed the colocalization of AKT (green) and RTKN (red) in HCC-LM3 and MHCC-97H cells. Scale bar, 20 μm. **C** HCC cells stably transduced with negative control or RTKN plasmids and lysates were immunoprecipitated with AKT and immunoblotted for p-AKT^473^, p-AKT^308^, GSK3β, p-GSK3β and AKT. **D–G** The protein expression of AKT, phosphorylated AKT, GSK3β and p-GSK3β was assessed in the indicated cell lines. *p < 0.05; **p < 0.01; ***p < 0.001. The data are shown as the means ± SEMs.

To further explore the impact of RTKN on the transcriptional regulation of β-catenin, we conducted immunoprecipitation using RTKN as a probe, followed by mass spectrometry (MS) analysis. The MS results identified AKT as an interacting protein (Fig S5B). Subsequent coimmunoprecipitation assays confirmed the interaction between AKT and RTKN (Fig. 6A). Immunofluorescence colocalization revealed that both proteins were present in the cytoplasm and the nucleus (Fig. 6B). Western blot analysis revealed that phosphorylated AKT (p-AKT) levels were elevated in RTKN-overexpressing cells, accompanied by an increase in phosphorylated GSK3β (p-GSK3β) levels. (Fig. 6C). These findings suggest that RTKN activates the AKT signaling pathway. To evaluate whether AKT contributes to tumor progression through the PI3K/AKT pathway, we treated RTKN-overexpressing cells with LY294002, a PI3K inhibitor. Prior to treatment, LY294002 administration did not alter RTKN expression levels (Fig. 6D, E). Western blot analysis revealed that the elevated levels of phosphorylated AKT and phosphorylated GSK3β in RTKN-overexpressing cells were effectively reduced upon additional treatment with LY294002 (Fig. 6D, E). Moreover, these experiments further revealed that PSMD1 knockdown suppressed AKT phosphorylation, an effect that was reversed by RTKN overexpression (Fig. 6F, G). Overall, PSMD1 may influence AKT and GSK3β phosphorylation by modulating RTKN ubiquitination and protein interactions. These results highlight the role of PSMD1 in regulating cell proliferation and apoptosis in HCC, likely via the β-catenin pathway.

### PSMD1 promotes the growth of HCC in vivo

**Fig. 7:**
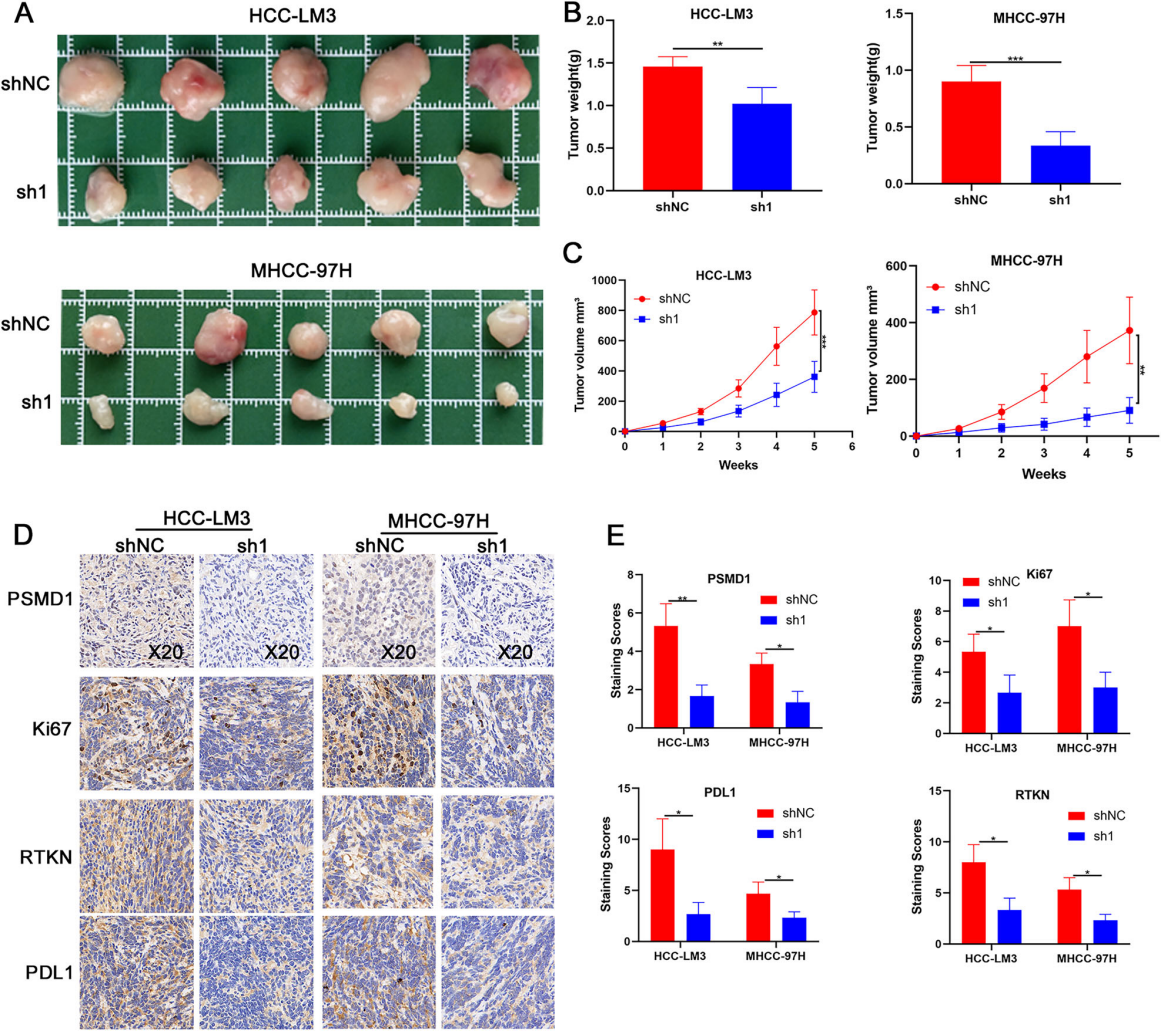
PSMD1 promotes the growth of HCC in vivo. **A** PSMD1 knockdown suppressed the growth of HCC xenografts in nude mice. **B**, **C** Tumor size and weight of the subcutaneous tumor growth model injected with HCC cells (5 mice/group). **D** IHC staining of the indicated proteins in tumor samples obtained from the subcutaneous tumor growth model. **E** IHC staining scores of xenograft tumors. *p < 0.05; **p < 0.01; ***p < 0.001. The data are shown as the means ± SEMs.

To investigate the tumor-promoting function of PSMD1 in HCC in vivo, we generated subcutaneous tumor xenograft models in BALB/c nude mice via PSMD1-knockdown MHCC-97H and HCC-LM3 cells. As shown in Fig. 7A–C, PSMD1 knockdown significantly inhibited tumor growth and decreased tumor weight in the xenograft mouse models. To further confirm the in vivo effects of PSMD1 knockdown, immunohistochemistry (IHC) analysis was performed. Compared with control tumors, PSMD1-knockdown tumors presented a marked reduction in Ki67 expression, indicating a decrease in cell proliferation. Additionally, the IHC results demonstrated a substantial decrease in RTKN and PD-L1 protein expression following PSMD1 knockdown (Fig. 7D, E). Collectively, these findings highlight the inhibitory effect of PSMD1 knockdown on tumor growth in vivo.

### Low expression of PSMD1 enhanced the antitumor efficacy of anti-PD1 therapy in vivo

**Fig. 8:**
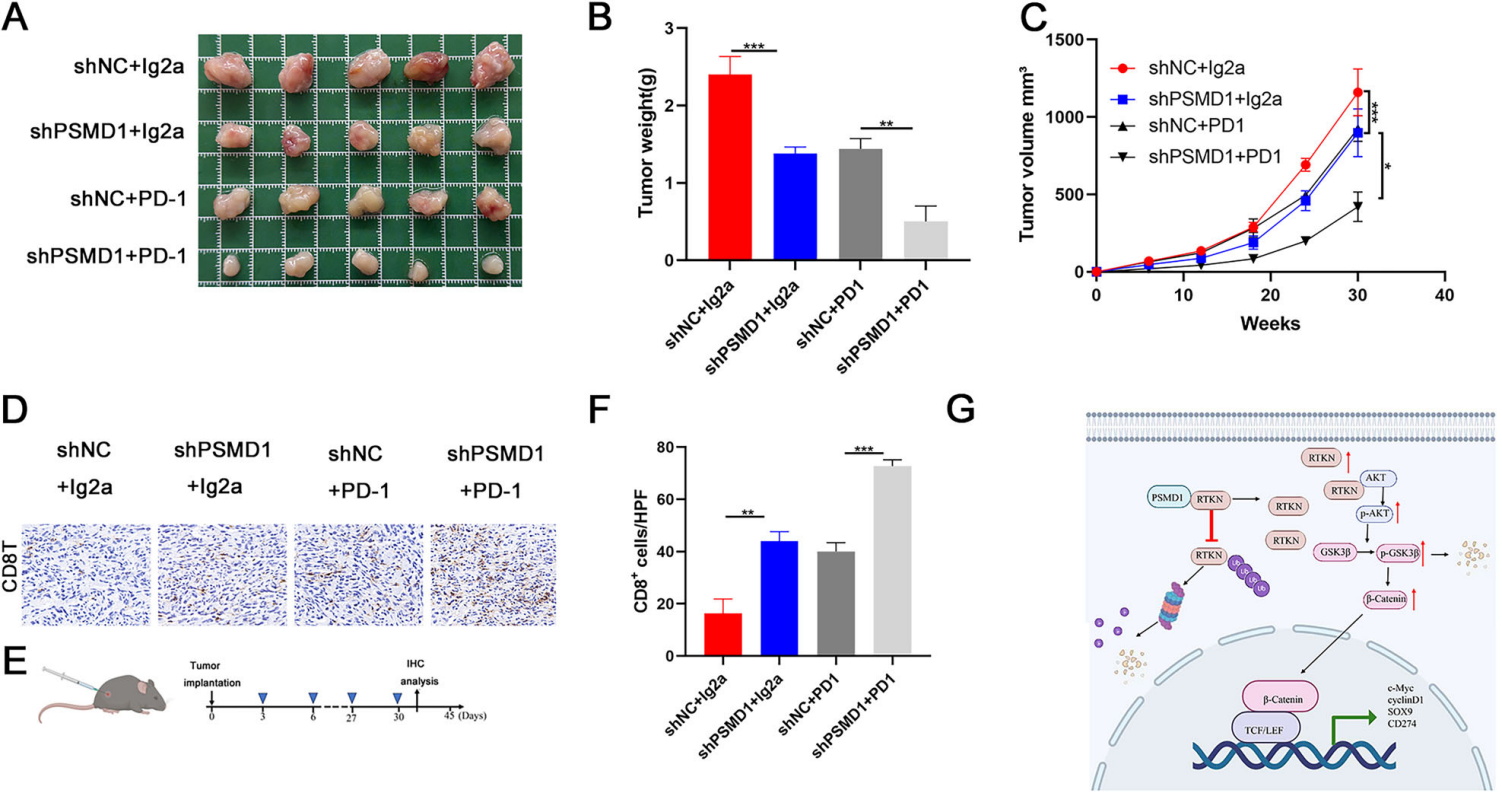
Low expression of PSMD1 enhanced the antitumor efficacy of anti-PD1 therapy in vivo. **A** Photographs of mouse tumors from each group (5 mice/group). **B**, **C** Tumor size and weight of the subcutaneous tumor growth model injected with HCC cells (5 mice/group). **D** IHC staining showing CD8 expression in HCC tissues. **E** Flow chart of the animal model. **F** Percent of CD8+ cells detected via IHC staining. **G** Proposed working model. *p < 0.05; **p < 0.01; ***p < 0.001. The data are shown as the means ± SEMs.

To investigate whether PSMD1 knockdown enhances the sensitivity of HCC to anti-PD-1 immunotherapy, orthotopic HCC mouse models were established with PSMD1-knockdown or control Hep1-6 cells (Fig. S5C). The mice were treated with either an anti-PD-1 monoclonal antibody (mAb) or IgG as a control (Fig. 8A). Tumor growth was significantly inhibited by both PSMD1 knockdown and anti-PD-1 immunotherapy. Notably, the combination of these two treatments achieved the most pronounced therapeutic effect (Fig. 8A–C). Mechanistically, anti-PD-L1 therapy exerts its antitumor effects predominantly by enhancing T-cell functions. The efficacy of this combined treatment was further validated by CD8⁺ T-cell staining in xenograft tumor tissues (Fig. 8D–F). In summary, our findings provide strong evidence that PSMD1 knockdown potentiates the efficacy of anti-PD-1 immunotherapy in HCC, highlighting PSMD1 as a potential therapeutic target.

## Discussion

Cancer immunotherapy based on PD-1 blockade has revolutionized cancer treatment approaches and paradigms, as it targets a broader range of cancer types, exhibits fewer side effects compared to other cancer therapies, and achieves a longer duration of therapeutic response [16]. Targeted and immunotherapies are effective treatment approaches for HCC [17, 18]. However, the response rates are not very satisfactory [5, 19]. To identify therapeutic targets related to patient prognosis and treatment response, we utilized patient data from TCGA for machine learning analysis, aiming to discover new potential targets for targeted therapies. PSMD1, identified as the most significantly differentially expressed target, was chosen as the focus of this study. Notably, PSMD1 is highly expressed in HCC tumor tissues and is associated with larger tumor size, more advanced TNM stage, and higher Edmondson grade. Moreover, PSMD1 expression is negatively correlated with patient prognosis. These findings highlight the potential of PSMD1 as a prognostic biomarker for HCC. Single-cell sequencing data revealed that PSMD1 is predominantly localized in HCC cells. In both in vivo and in vitro models, PSMD1 knockdown significantly suppressed HCC growth and enhanced the efficacy of immunotherapy. Moreover, PSMD1 knockdown promoted increased infiltration of CD8^+^ T cells. Collectively, our findings demonstrate that PSMD1 knockdown inhibits HCC proliferation, induces apoptosis, and enhances the therapeutic efficacy of PD-1 blockade, highlighting PSMD1 as a promising target for therapeutic intervention (Fig. 8G).

Furthermore, β-catenin plays a crucial role in maintaining stem cell characteristics, driving invasiveness, and enabling immune evasion [20, 21]. In tumor cells, elevated expression of β-catenin can prevent spontaneous T-cell activation and infiltration into the tumor microenvironment, thereby contributing to resistance against immune checkpoint inhibitors, such as PD-1 and CTLA4 therapies. In tumor models such as melanoma, breast cancer, neuroblastoma, and renal adenocarcinoma mouse models, β-catenin blockade combined with immune checkpoint inhibitors has been shown to induce tumor regression [22, 23]. Therefore, exploring the molecular mechanisms that influence the β-catenin signaling pathway in the immunoregulation of HCC could provide valuable insights for advancing cancer immunotherapy. Bioinformatics analysis revealed that PSMD1 is overexpressed in HCC and is closely linked to the activation of β-catenin signaling. Knockdown of PSMD1 resulted in decreased expression levels of β-catenin and its downstream signaling pathway proteins. Rescue experiments using a β-catenin agonist revealed that PSMD1 exerts its biological functions in a β-catenin-dependent manner. These findings suggest that PSMD1 may play a pivotal role in the dysregulation of β-catenin signaling.

Rhotekin (RTKN) is the gene encoding the Rho effector. The involvement of RTKN in cancers has recently been investigated. High expression of RTKN has been reported in several cancers [14, 15, 24]. For example, in gastric cancer, RTKN promotes NF-κB activation, thereby inhibiting tumor cell apoptosis and facilitating tumor progression [25]. The molecular mechanisms underlying the development and progression of RTKN in HCC remain incompletely understood. Mass spectrometry analysis demonstrated that PSMD1 binds to RTKN, preventing its ubiquitination-mediated degradation. Consistent with previous studies implicating RTKN in β-catenin activation, our mass spectrometry analyses revealed that RTKN interacts with AKT and promotes its activation, as evidenced by phosphorylation at Thr308 and Ser473, the canonical markers of full AKT activation. This, in turn, facilitates the phosphorylation of GSK3B, leading to increased β-catenin protein levels and increased PD-L1 transcription. Thus, our study reveals the molecular mechanism underlying tumor PD-L1 regulation.

While our study revealed the role of PSMD1 in HCC proliferation and its function in immunotherapy, the posttranslational modifications of PSMD1 and their impact on the inhibition of RTKN ubiquitination-mediated degradation remain unclear. In addition, the translational relevance of these findings warrants further investigation, for example, using patient-derived organoid models and clinical trials.

## Conclusion

We identified a novel expression profile of PSMD1 in HCC and demonstrated that PSMD1 regulates immune reprogramming. These findings suggest that the PSMD1/RTKN/β-catenin/PD-L1 signaling axis may serve as a promising therapeutic target for HCC.

## Materials and methods

### Tissues and cell lines

One hundred paired samples of hepatocellular carcinoma (HCC) tissues and adjacent normal liver tissues were intraoperatively collected from patients at the First Affiliated Hospital of Nanjing Medical University. Patients with a history of antitumor therapies were excluded from the study. All patients provided written informed consent. The research was conducted with the approval of the hospital’s ethics committee.

The HCC cell lines used in this study were sourced from the Cell Bank of Type Culture Collection (Shanghai, China), while primary human hepatocytes were isolated from liver samples obtained during hepatectomy via a modified two-step collagenase perfusion method [26]. All the cell lines were cultured in Dulbecco’s modified Eagle’s medium (DMEM) supplemented with 10% fetal bovine serum (FBS; Gibco, NY, USA) and maintained under standard conditions (37 °C, 5% CO₂).

### Statistical analysis

In this study, numerical data and histograms are presented as the means ± SEMs, with detailed statistical parameters provided in the figure legends. Each experiment was performed at least three times. The data were analyzed via Prism 8 software (GraphPad Software, La Jolla, CA, USA). Group differences were assessed with Student’s *t* test. Comparisons of more than two groups were done with two-way ANOVA and Tukey’s multiple comparison correction. For survival analysis, overall survival (OS) rates were calculated via the Kaplan‒Meier method and compared via the log-rank test. Statistical significance was defined as p < 0.05, p < 0.01, and p < 0.001, indicating the levels of significance in the results.

Details on other materials and methods are provided in the supplementary materials.

## Supplementary information

Supplemental material figure legends

Supplementary materials and methods

the original Western blots

Figure S1

Figure S2

Figure S3

Figure S4

Figure S5

Table S1

Table S2

Table S3

Table S4

Table S5

## Data Availability

All the data generated or analyzed during this study are included in the additional files.
