# Supplementary material for: PSMD1 inhibition suppresses tumor progression and enhances antitumor immunity by modulating the RTKN/β-catenin/PD-L1 axis in hepatocellular carcinoma
Source: Cell Death Dis. 2026 Jan 14;17(1):36. doi: 10.1038/s41419-025-08241-4 (PMC12804919; doi:10.1038/s41419-025-08241-4)
Supplement: Supplementary file 1 — Supplemental material figure legends [file 41419_2025_8241_MOESM1_ESM.docx]

**Figure S1. PSMD1 is an oncogene associated with HCC progression and clinical outcomes. A-B**. LASSO regression coefficients (A) and partial likelihood deviance (B) under diverse log λ values. **C.** Comparison of minimal depth and VIMP rankings in the random survival forest (RSF). **D-E.** Kaplan–Meier curves of OS according to the risk score in the training (D) and validation (E) cohorts. **F-H.** Comparison of the ESTIMATE score (F), immune score (G) and stromal score (H) for tumors in the high- and low-risk groups. **I.** The distribution of infiltration of 28 immune cell subsets inferred by CIBERSORT between the high- and low-risk groups. **J.** Scatter plot showing the correlation between the expression of PSMD1 and that of PD-L1. **K.** Knockdown of PSMD1 affected the protein levels of Bcl2 and BAX in HCC cells. *p<0.05; **p<0.01; ***p<0.001. The data are shown as the means ± SEMs.

**Figure S2.** **PSMD1 promotes tumor proliferation through the β-catenin pathway. A.** The luciferase activity of β-catenin/Tcf4 transcriptional activity was measured in shPSMD1 and control HCC cells with or without SKL2001. **B.** The protein levels of CyclinD1, c-Myc, SOX9, AXIN2, ABCG2, c-jun and PD-L1 in HCC cells. **C** Colony formation assay data for HCC cells. **D.** EdU assay data for HCC cells. **E.** CCK-8 assay data for HCC cells. **F, G.** EdU and colony formation assays of HCC cells. *p<0.05; **p<0.01; ***p<0.001. The data are shown as the means ± SEMs.

**Figure S3. PSMD1 inhibits apoptosis through the β-catenin pathway. A, B.** Cell apoptosis assays for HCC cells. **C.** Mass spectrogram of the RTKN protein**. D.** A co-IP assay was performed in HCC cells. **E.** Immunofluorescence analysis revealed the colocalization of RTKN (green) and PSMD1 (red) in MHCC-97H cells. Scale bar, 20 μm. **F, G.** Protein and mRNA expression levels of RTKN and PSMD1 in HCC cells. * p<0.05; **p<0.01; ***p<0.001. The data are shown as the means ± SEMs.

**Figure S4. PSMD1 regulates the progression of HCC in an RTKN-dependent manner. A.** RTKN expression in the TCGA-LIHC cohort. **B.** Kaplan–Meier curves of OS in the TCGA-LIHC cohort. **C.** The protein levels of CyclinD1, c-Myc, SOX9, AXIN2, ABCG2, c-jun and PD-L1 in HCC cells. **D-H.** Colony formation and EdU assays for HCC cells. **I-J.** Cell apoptosis assays for HCC cells. *p<0.05; **p<0.01; ***p<0.001. The data are shown as the means ± SEMs.

**Figure S5. RTKN binds to AKT in HCC cells**. A. CCK-8 assay data for HCC cells. B. Mass spectrogram of the AKT protein. C. mRNA expression levels of PSMD1 in HCC cells. *p<0.05; **p<0.01; ***p<0.001. The data are shown as the means ± SEMs
