# Supplementary material for: PSMD1 inhibition suppresses tumor progression and enhances antitumor immunity by modulating the RTKN/β-catenin/PD-L1 axis in hepatocellular carcinoma
Source: Cell Death Dis. 2026 Jan 14;17(1):36. doi: 10.1038/s41419-025-08241-4 (PMC12804919; doi:10.1038/s41419-025-08241-4)
Supplement: Supplementary file 2 — Supplementary materials and methods [file 41419_2025_8241_MOESM2_ESM.docx]

**Materials and methods**

**Animal experiments**

Four-week-old male nude mice and C57/B6 mice were obtained from the Animal Core Facility of Nanjing Medical University (Nanjing, China) for in vivo tumor growth experiments. The mice were randomly divided into four groups, with five mice per group.

A total of 2×10^6^ MHCC-97H or HCC-LM3 cells with stable PSMD1 knockdown were suspended in 100 μL of PBS and injected subcutaneously into the dorsal flanks of the mice. Tumor volumes and weights were recorded at specific time points. At the endpoint, the samples were fixed in 4% paraformaldehyde and analyzed via immunohistochemistry (IHC).

For the immune-competent mouse model, shPSMD1 Hepa1-6 cells were inoculated to assess the role of an intact immune system in the PSMD1-induced immune response. A mouse PD-1 monoclonal antibody (mAb; BioXcell, BE0146) and an IgG isotype control (BioXcell, BE0089) were administered to evaluate the potential synergistic effect of shPSMD1 and PD-1 mAb therapy.

**Transfection experiment**

To establish stably transfected cell lines, lentiviral vectors for shPSMD1 knockdown and RTKN overexpression, along with their respective controls, were obtained from Genechem (Shanghai, China). The sequences of the shRNA oligonucleotides are provided in Supplementary Table 4.

**QRT‒PCR**

RNA was extracted from both HCC tissues and cell lines via TRIzol reagent (Invitrogen, USA) following established protocols (1). The isolated RNA was then reverse transcribed to generate complementary DNA (cDNA), which was subsequently used as a template for RT‒qPCR analysis. Human GAPDH gene expression served as an internal reference to normalize the mRNA levels. Relative gene expression was calculated via the 2−ΔΔCt method. The specific sequences of primers used in this study are listed in Supplementary Table S2.

**Western blot analysis**

Proteins were extracted via RIPA buffer (Beyotime, Shanghai, China) supplemented with protease inhibitors. The lysates were separated on 10% SDS‒PAGE gels and transferred to PVDF membranes (Millipore, MA, USA). The membranes were blocked for 50 minutes with fast blocking buffer (Beyotime, Shanghai, China) and incubated overnight at 4°C with primary antibodies. After incubation with secondary antibodies for 1 h, the membranes were washed three times (10 min each) in TBST (Tris-buffered saline with Tween-20). Protein signals were detected via an enhanced chemiluminescence (ECL) Western Blotting Kit (Millipore, MA, USA). The details of the antibodies used are listed in Table S3.

**Nuclear protein extraction assay**

The experiments followed the protocol provided by the manufacturer for the Qproteome Cell Compartment Kit (Qiagen). For each sample, 20 μg of protein was loaded per well. SDS‒PAGE (10%) and western blotting were conducted as previously described.

**Cycloheximide (CHX) treatment**

HCC cells were treated with cycloheximide (CHX, 25 μg/mL; Selleck, China), a protein synthesis inhibitor, for various durations (0, 2, 4, and 8 hours). After treatment, proteins were extracted and analyzed via western blotting to evaluate the impact of CHX on protein expression over time.

**Chemicals**

The PI3K inhibitor LY294002 (HY-10108) and the β-catenin agonist SKL2001 from MedChemExpress were both dissolved in DMSO, with working concentrations of 20 μM (2) and 40 μM(3), respectively.

**CCK-8 assay**

The Cell Counting Kit-8 (CCK-8) assay was performed as previously described (1).

**Colony formation assay**

For the colony formation assay, 600 cells per well were seeded in 6-well plates and cultured in complete medium for 10 days. Colonies were fixed with formaldehyde and stained with 0.1% crystal violet (Vicmed, China). The stained colonies were photographed and manually counted.

**EdU assay**

The EdU proliferation assay was conducted using an EdU kit (Beyotime, Shanghai, China). The cells were seeded at a density of 1 × 10⁴ cells per well in 96-well plates and cultured for 12 hours. Following a 2-hour EdU incubation, subsequent steps were performed according to the manufacturer’s protocol. After DAPI staining (Beyotime, Shanghai, China), the nuclei were visualized with an inverted fluorescence microscope (Nikon, Tokyo, Japan).

**Co‑immunoprecipitation (co‑IP) assay**

To assess the interaction between RTKN and PSMD1, as well as RTKN and AKT proteins, immunoprecipitation was performed via an IP kit (Thermo Fisher Scientific, CA, USA) following the manufacturer’s guidelines. The precipitated proteins were subsequently analyzed by immunoblotting.

**Flow cytometry assay**

Apoptosis analysis of the cells was conducted following our previously described method (1).

**IHC**

Tumor tissues were fixed in 4% paraformaldehyde, dehydrated with a graded ethanol series, embedded in paraffin, and sectioned into 4 μm slices. The sections were treated with 3% H₂O₂ for 20 minutes to block endogenous peroxidase activity, followed by a 30-minute incubation with a blocking solution. The sections were subsequently incubated overnight at 4°C with primary antibodies. The remaining steps were performed as previously described. Images were captured via a microscope (Leica Microsystems, Germany), and the staining results were independently evaluated in a double-blinded manner.

**Immunofluorescence staining**

Immunofluorescence analysis was conducted to determine protein localization and expression within cells. The cells were cultured in confocal dishes, fixed with 4% paraformaldehyde, and washed with PBS. Permeabilization was performed with 0.5% Triton X-100 for 20 minutes. After being blocked with 10% goat serum at room temperature (RT) for 30 minutes, the cells were incubated overnight at 4°C with primary antibodies.

The following day, the cells were washed with PBST and incubated with fluorescence-conjugated secondary antibodies for 1 hour at 37°C. Nuclei were counterstained with DAPI for 10 minutes at RT, and the cells were mounted with glycerol. Finally, images were acquired via a confocal microscope (FV3000, Olympus).

**Publicly available data collection and processing**

The RNA-Seq data of hepatocellular carcinoma were accessed from The Cancer Genome Atlas (TCGA) database (https://portal.gdc.cancer.gov/projects/TCGA-LIHC) and International Cancer Genome Consortium (ICGC) database (https://dcc.icgc.org/releases/current/Projects/LIRI-JP), respectively. Transcriptome profiling data were obtained and normalized by log2(1+TPM), where TPM denoted transcripts per million. After acquiring normalized data and excluding samples without survival information, we extracted 368 patients from the TCGA and 240 patients from the ICGC dataset as external validation. Immune-related genes were obtained from the import database (https://www.immport.org/shared/), and we established a 1793-gene immune genes set after deduplication.

**Comprehensive Screening and Identification of Prognostic Genes**

Firstly, the TCGA cohort was randomly split into a training cohort and a validation cohort at a 7:3 ratio, while the ICGC cohort was used as an external validation set. To develop an accurate and stable immune-related prognostic signature, we integrated random survival forest (RSF), least absolute shrinkage and selection operator (LASSO), and Cox proportional regression.

Initially, univariate Cox regression using the R package ezcox identified prognostic genes in the training cohort. The R package glmnet was then employed to perform LASSO Cox regression with 10-fold cross-validation to screen candidate biomarkers. RSF analysis, using R package randomForestSRC, assessed gene importance in predicting overall survival (OS) of HCC patients through the Variable Importance (VIMP) and minimal depth methods. Genes identified by both LASSO and RSF were further analyzed via multivariate Cox regression to construct a prognostic gene signature as follows: Riskscore = ∑_(i=1)^n▒〖βi*Expi〗 (Expi represents the expression of the i gene, and βi represents the coefficient of the i gene). Genes identified by LASSO: “BLNK, C3, FGF19, GLP1R, HSP90AA1, IL15RA, IL18RAP, MAPT, NR1H3, S100A9, SLC29A3, SSTR2, STC1, TMPRSS6, TXLNA, EPO, FGF9, PSMD1”. Genes identified by RSF: “EPO, FGF9, PSMD1, PPIA, CHP1, PSMD14, HSPA4, CACYBP, NDRG1”. Based on the median risk score, patients were classified into high- and low-risk groups, and Kaplan–Meier survival analysis was conducted to assess survival probabilities in each cohort.

**Immune infiltration and immunotherapy Analysis between two risk groups**

ESTIAMTE algorithm (Estimation of Stromal and Immune cells in Malignant Tumour tissues) was employed to generate immune and stromal scores based on RNA-seq data. Additionally, to assess the relative proportions of 22 infiltrating immune cell types, the CIBERSORT algorithm was utilized. Differences in infiltration scores between high-risk and low-risk groups were then analyzed using the t-test, or the Mann-Whitney test when non-parametric.

TIDE scores were calculated for each sample using the TIDE database (http://tide.dfci.harvard.edu/) to evaluate immunotherapy efficacy.

**Single-cell RNA-sequencing Analysis**

For analysis of single-cell RNA-sequencing (scRNA) data, the GSE149614 dataset for HCC was obtained from Gene Expression Omnibus (GEO) database (https://www.ncbi.nlm.nih.gov/geo/query/acc.cgi?acc=gse149614), including 10 patients with primary tumors and 8 patients with normal liver tissue, and subsequent analyses were performed. Single-cell data were processed using the Seurat package (version 5.1.0). The data were normalized, and 2,000 highly variable genes were identified. Principal component analysis (PCA) was performed on these genes, with the top 20 principal components (PCs) selected. The Harmony package was then applied for multi-group data integration, followed by dimensionality reduction and cell clustering using uniform manifold approximation and projection (UMAP). Cells with mitochondrial gene expression below 10% and between 200 and 8,000 detected genes were retained, resulting in 49,431 cells after filtration. Then, cell‐cell communication analysis was conducted using the published R package CellChat.

**Gene Set Enrichment Analysis (GSEA)**

GSEA is commonly used to determine whether a predefined set of genes shows statistically significant differences between two biological data sets. In this study, GSEA was employed to identify major activated pathways in the high risk group.

1. Hao X, Zhang Y, Shi X, Liu H, Zheng Z, Han G, et al. CircPAK1 promotes the progression of hepatocellular carcinoma via modulation of YAP nucleus localization by interacting with 14-3-3zeta. J Exp Clin Cancer Res. 2022;41(1):281.

2. Chen QT, Zhang ZY, Huang QL, Chen HZ, Hong WB, Lin T, et al. HK1 from hepatic stellate cell-derived extracellular vesicles promotes progression of hepatocellular carcinoma. Nat Metab. 2022;4(10):1306-21.

3. Li B, Jiang T, Wang J, Ge H, Zhang Y, Li T, et al. Cuprorivaite microspheres inhibit cuproptosis and oxidative stress in osteoarthritis via Wnt/beta-catenin pathway. Mater Today Bio. 2024;29:101300.
