# Supplementary material for: PSMD1 inhibition suppresses tumor progression and enhances antitumor immunity by modulating the RTKN/β-catenin/PD-L1 axis in hepatocellular carcinoma
Source: Cell Death Dis. 2026 Jan 14;17(1):36. doi: 10.1038/s41419-025-08241-4 (PMC12804919; doi:10.1038/s41419-025-08241-4)
Supplement: Supplementary file 3 — the original Western blots [file 41419_2025_8241_MOESM3_ESM.pdf]

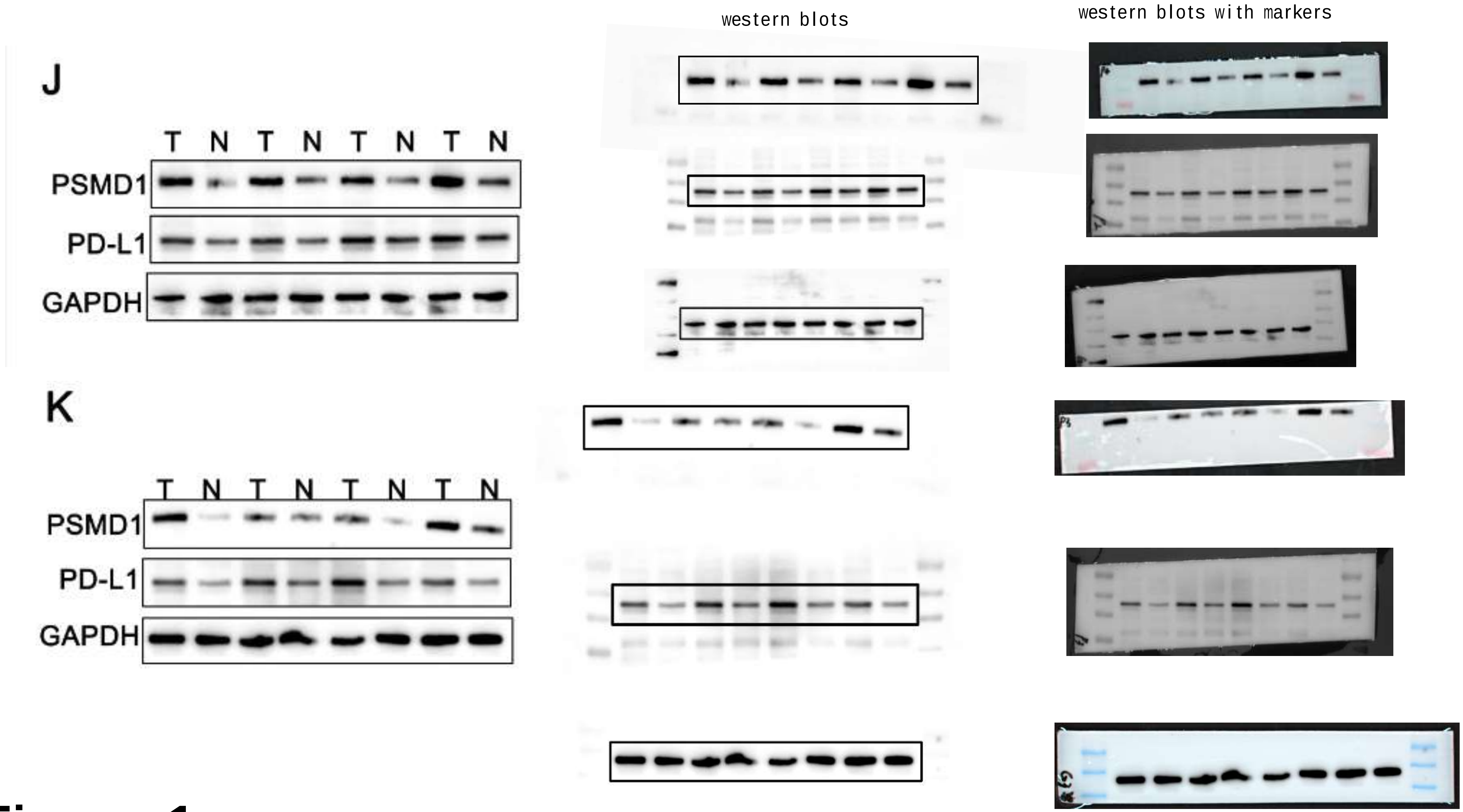
western blots
western blots with markers
J
T N T N T N T N
PSMD1
PD-L1
GAPDH
K
T N T N T N T N
PSMD1
PD-L1
GAPDH

Figure 1

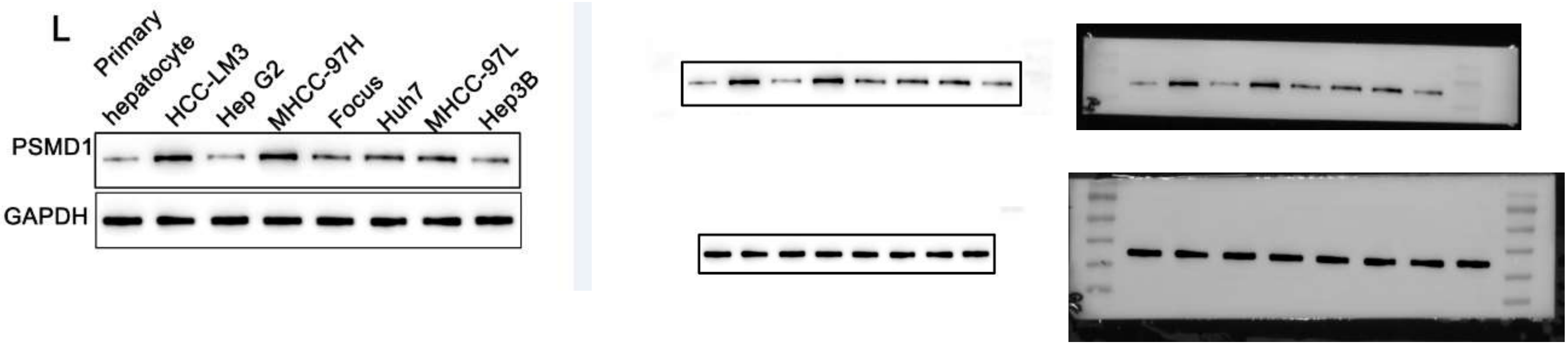
L
Primary hepatocyte
HCC-LM3
Hep G2
MHCC-97H
Focus
Huh7
MHCC-97L
Hep3B
PSMD1
GAPDH

Figure 1

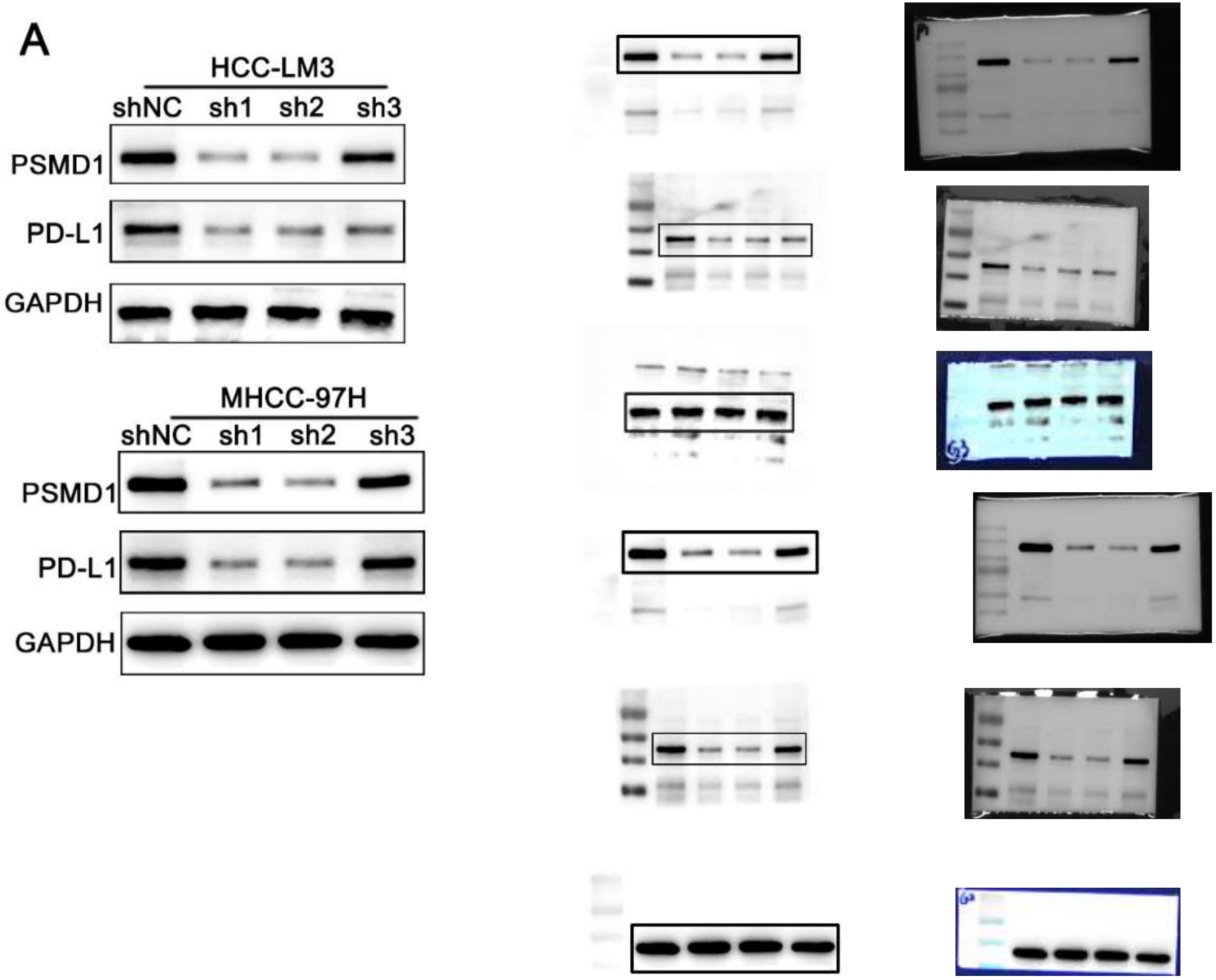
A
HCC-LM3
shNC sh1 sh2 sh3
PSMD1
PD-L1
GAPDH
MHCC-97H
shNC sh1 sh2 sh3
PSMD1
PD-L1
GAPDH

Figure 3

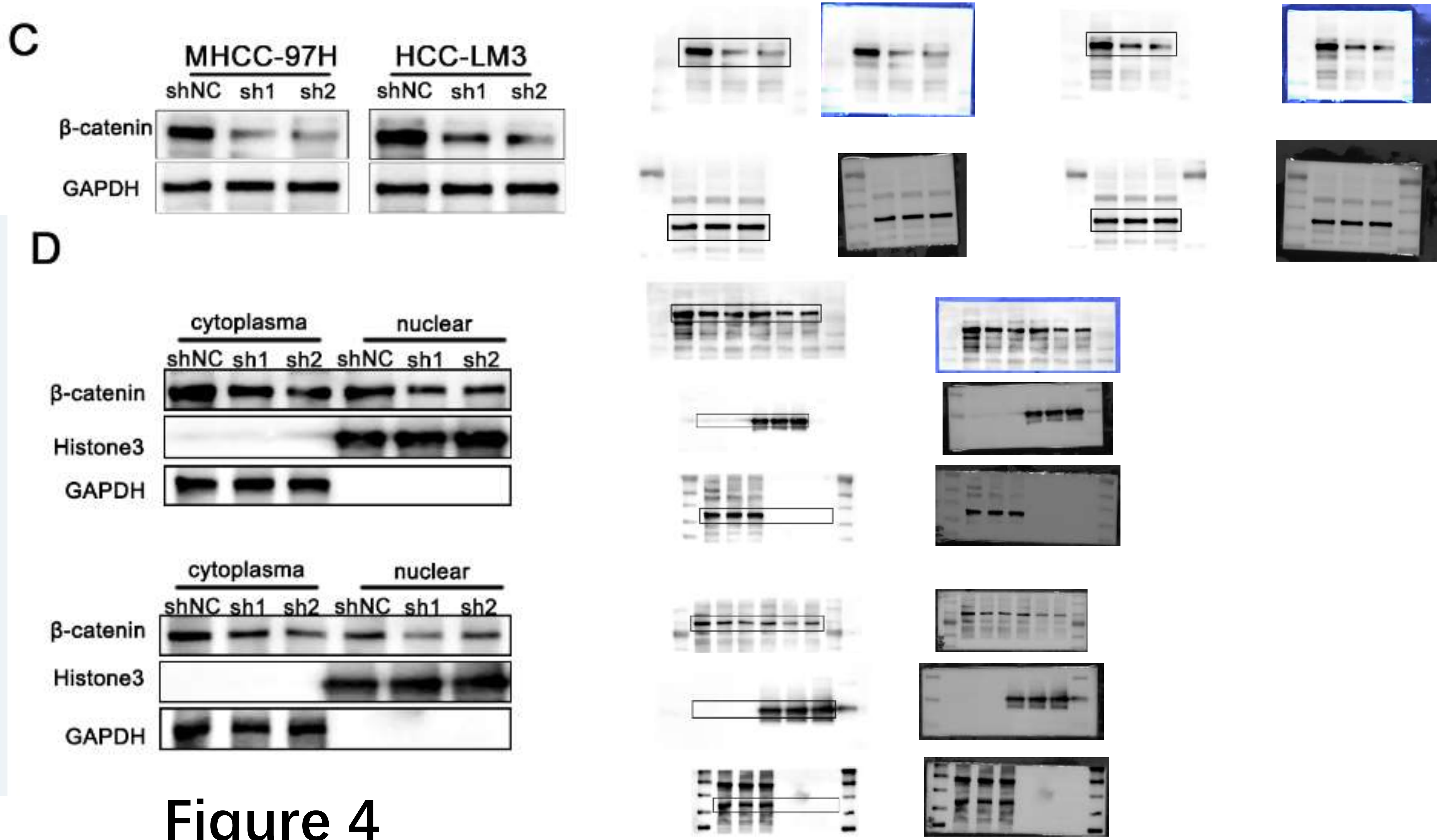
C
MHCC-97H
HCC-LM3
shNC sh1 sh2
shNC sh1 sh2
β-catenin
GAPDH
D
cytoplasma
nuclear
shNC sh1 sh2 shNC sh1 sh2
β-catenin
Histone3
GAPDH
cytoplasma
nuclear
shNC sh1 sh2 shNC sh1 sh2
β-catenin
Histone3
GAPDH

Figure 4

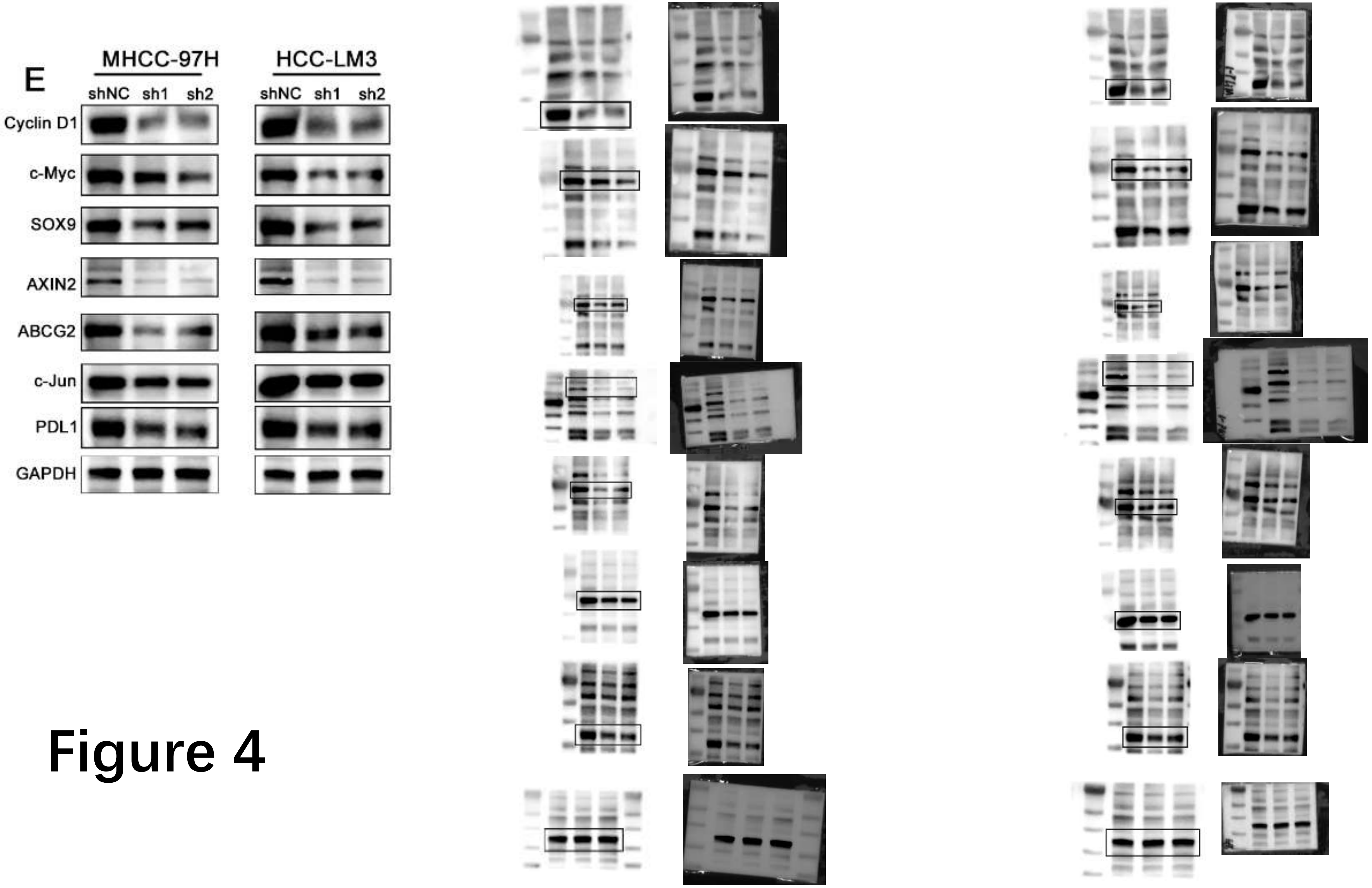
E
MHCC-97H
HCC-LM3
shNC
sh1
sh2
shNC
sh1
sh2
Cyclin D1
c-Myc
SOX9
AXIN2
ABCG2
c-Jun
PDL1
GAPDH

Figure 4

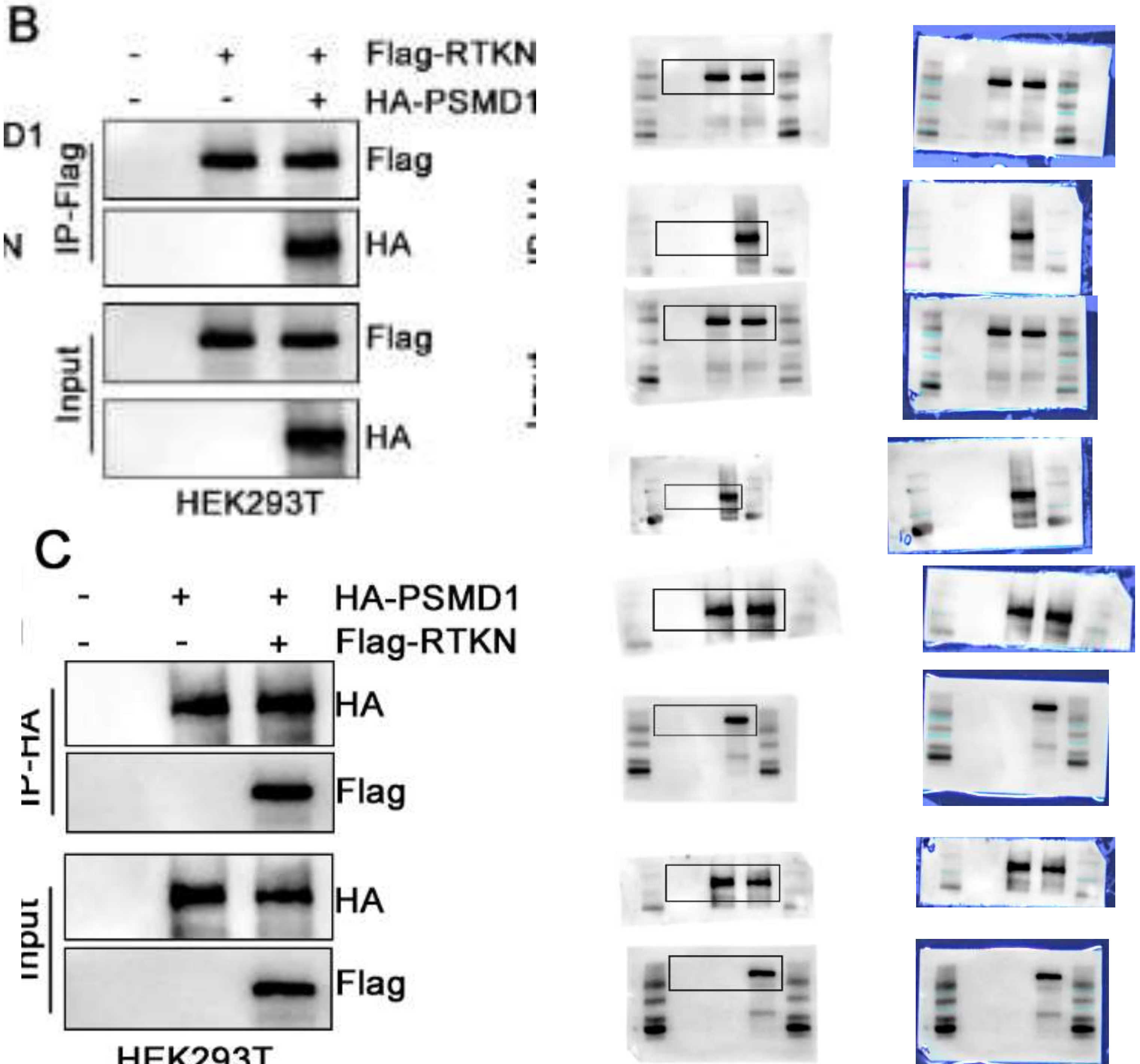
B
Flag-RTKN
HA-PSMD1
D1
N
IP-Flag
Flag
HA
Input
Flag
HA
HEK293T
C
HA-PSMD1
Flag-RTKN
HA
Flag
HA
Flag

Figure 5

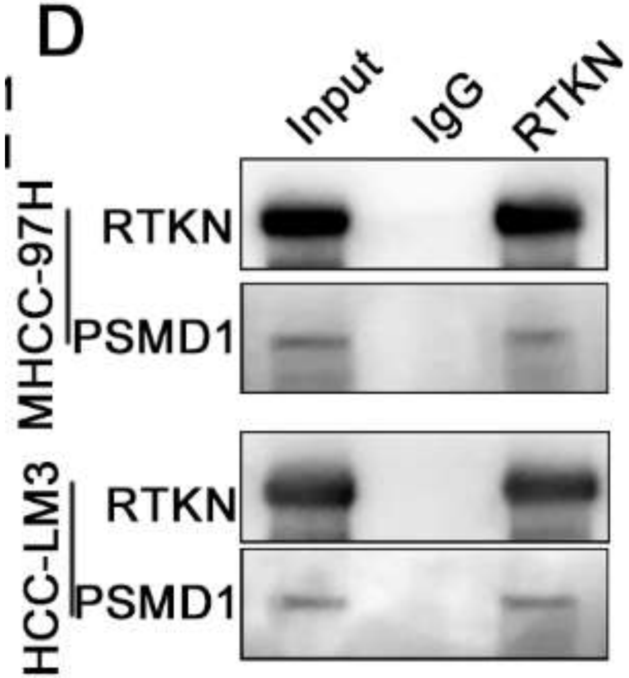

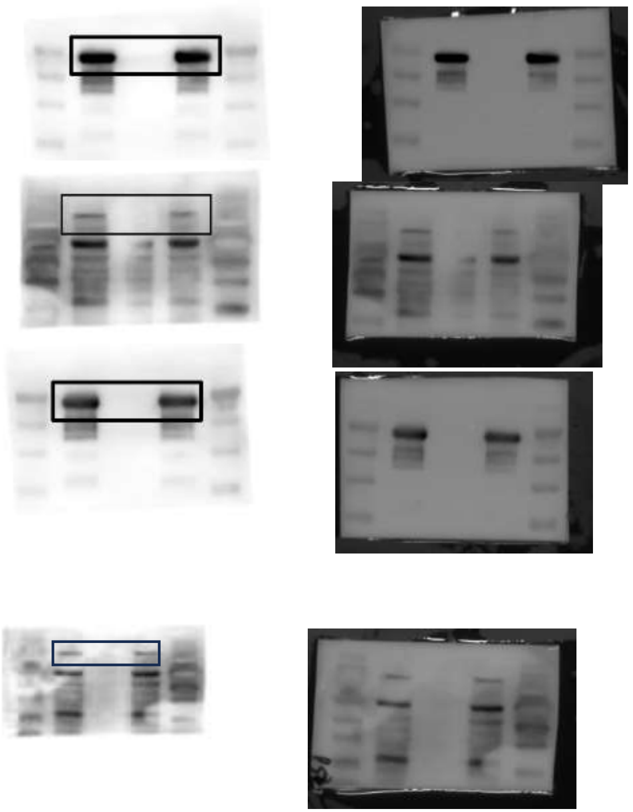

Figure 5

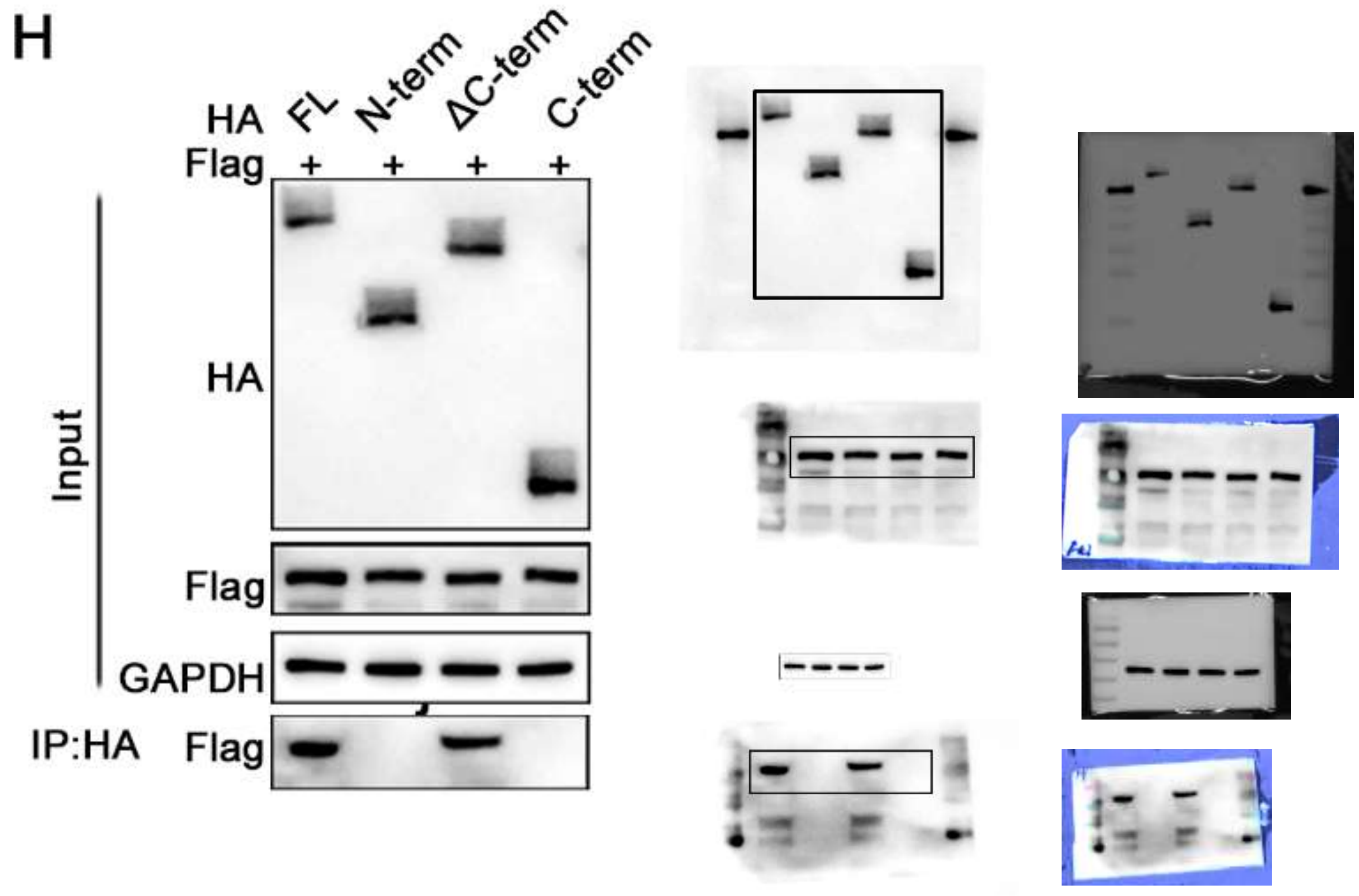

Figure 5

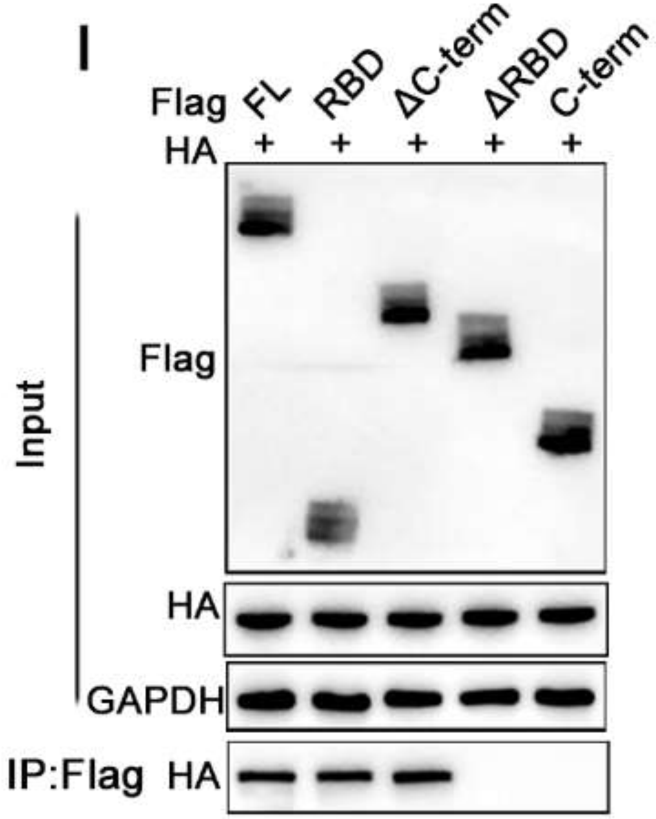
I
Flag
FL
RBD
ΔC-term
ΔRBD
C-term
HA
+
+
+
+
+
Input
Flag
HA
GAPDH
IP:Flag
HA

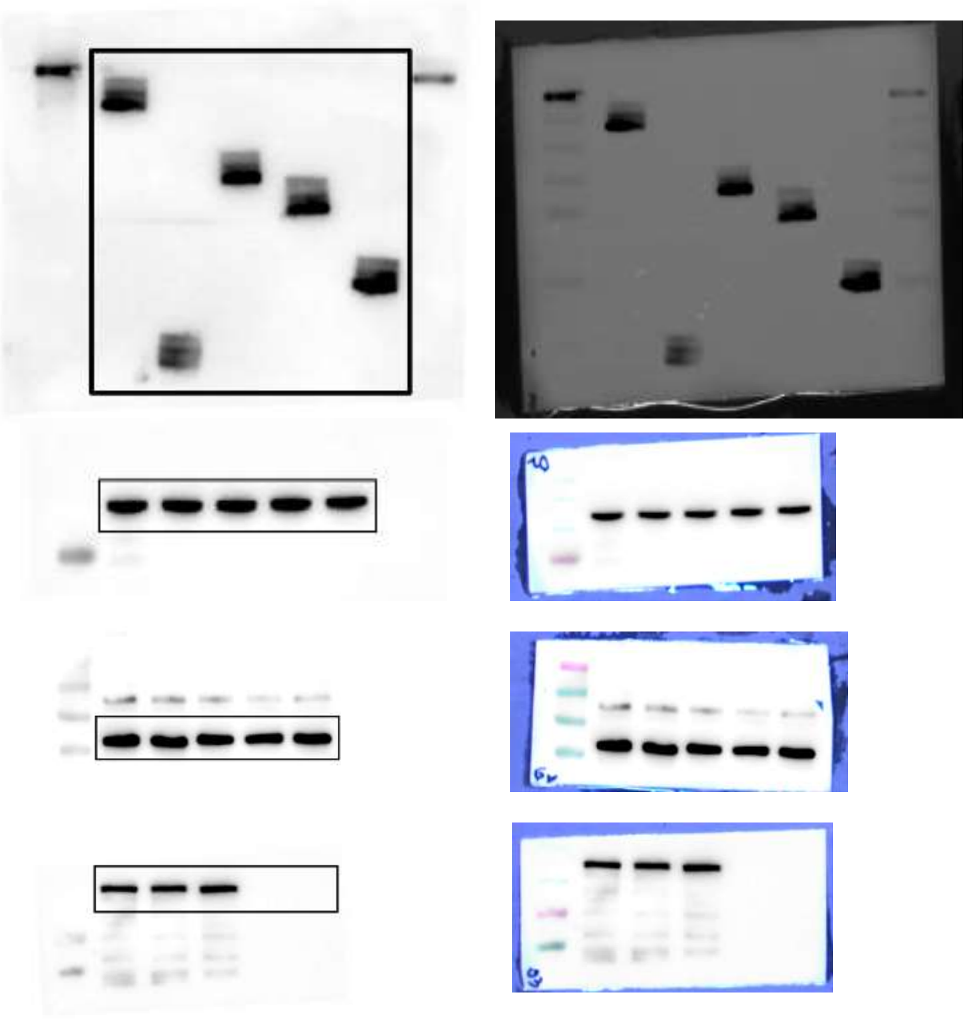

Figure 5

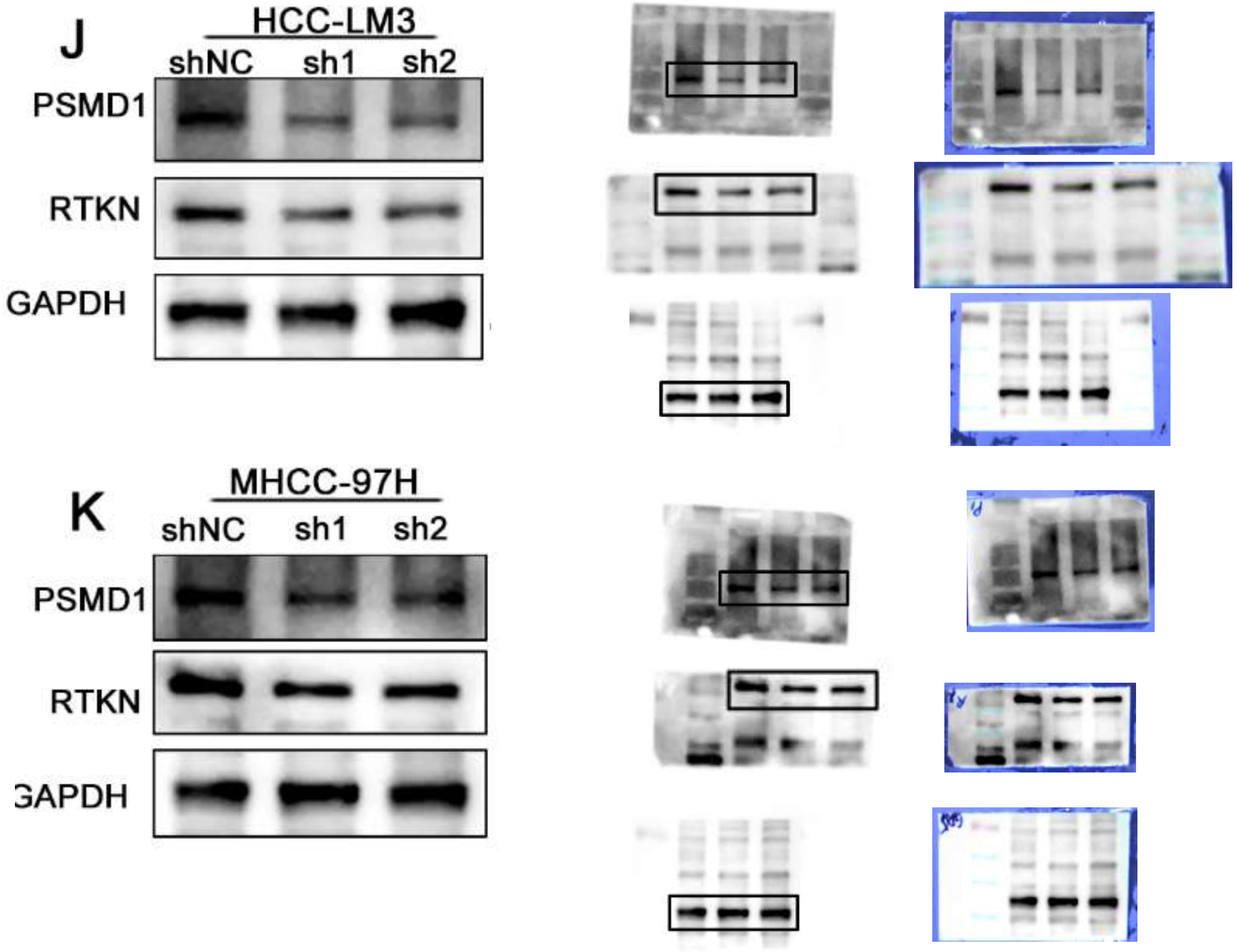
J
HCC-LM3
shNC
sh1
sh2
PSMD1
RTKN
GAPDH
K
MHCC-97H
shNC
sh1
sh2
PSMD1
RTKN
GAPDH

Figure 5

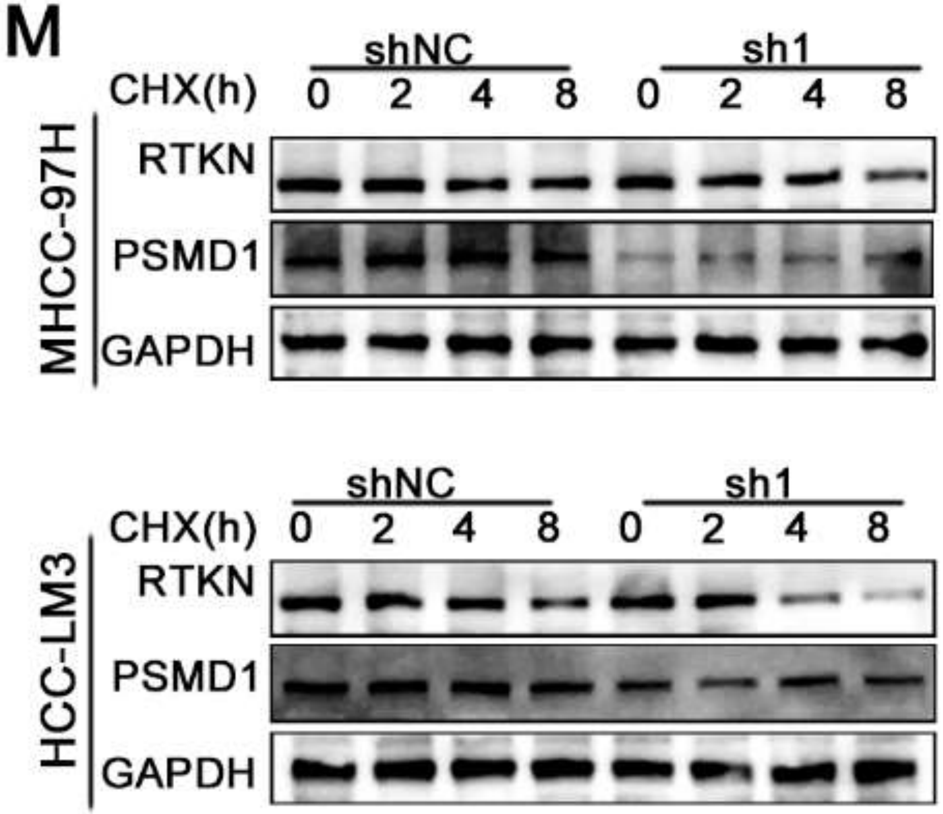
M
shNC
sh1
CHX(h)
0
2
4
8
0
2
4
8
MHCC-97H
RTKN
PSMD1
GAPDH
shNC
sh1
CHX(h)
0
2
4
8
0
2
4
8
HCC-LM3
RTKN
PSMD1
GAPDH

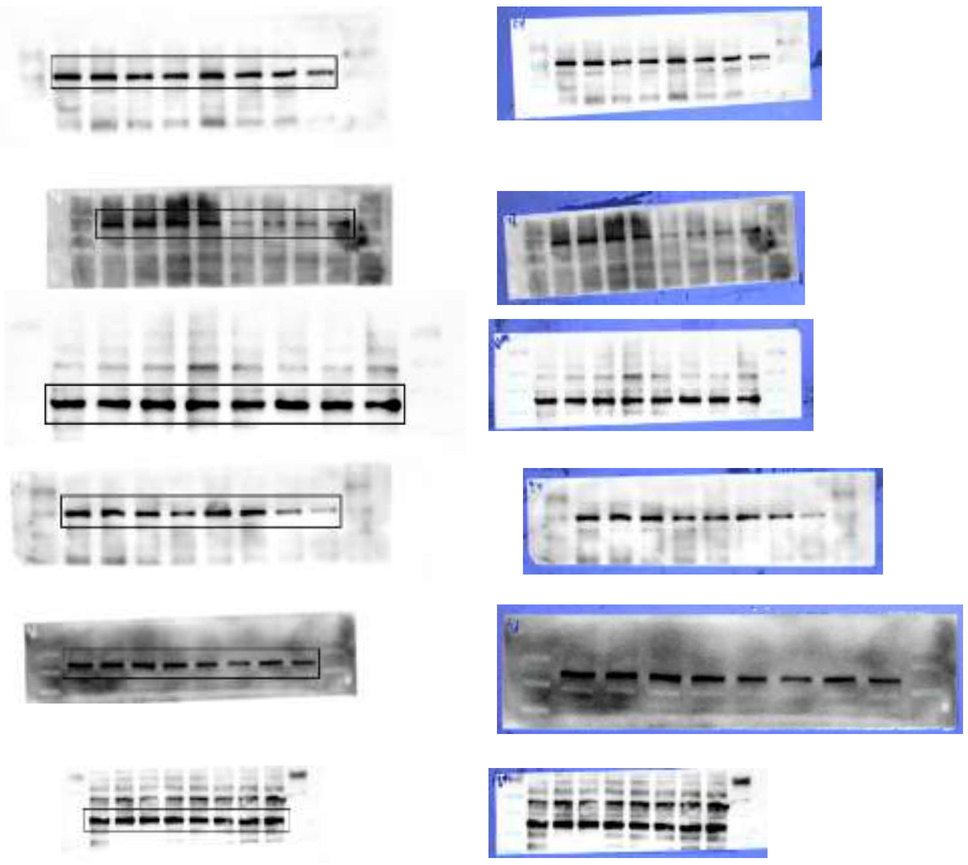

Figure 5

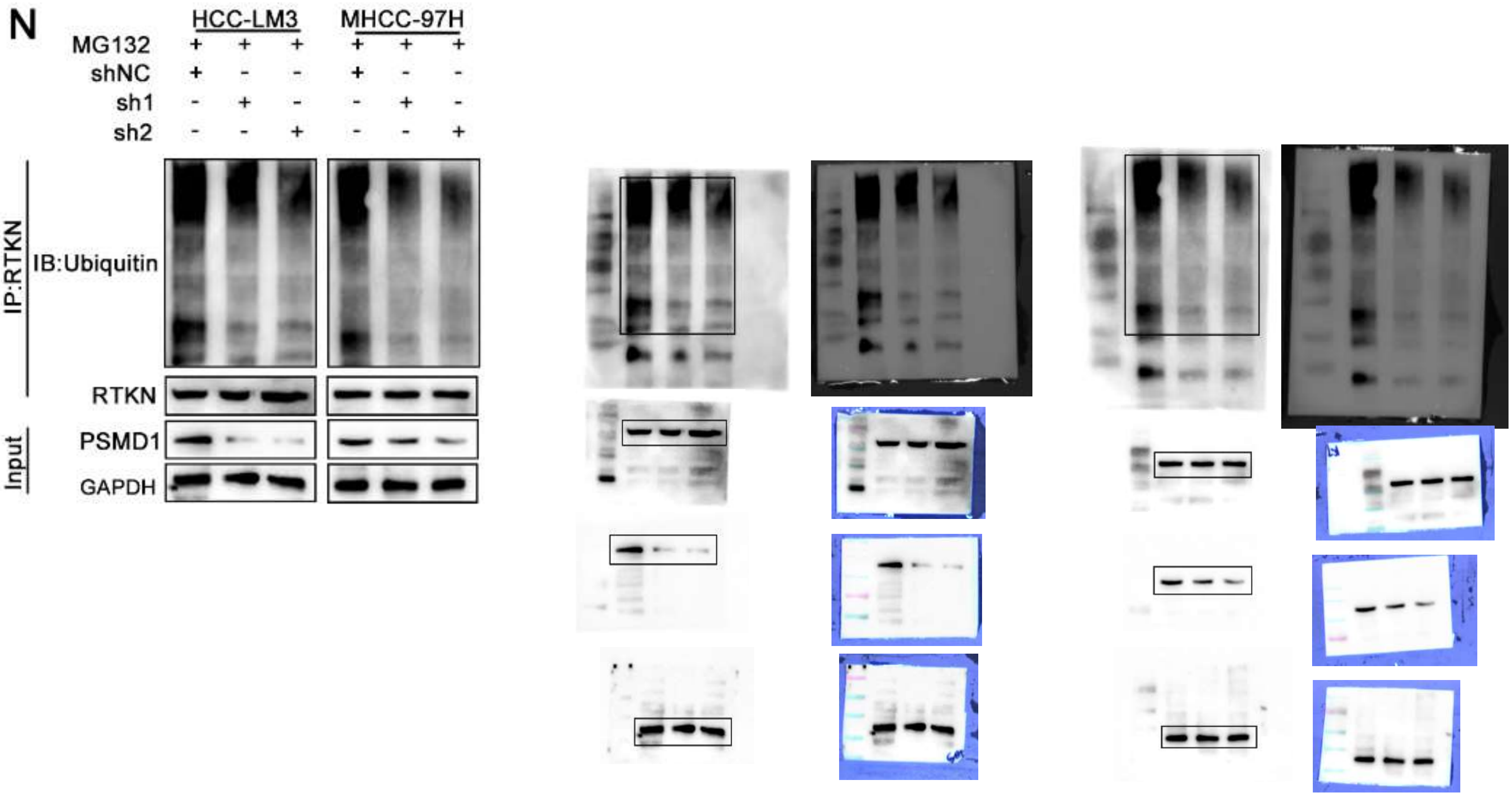
N
HCC-LM3
MHCC-97H
MG132
shNC
sh1
sh2
IP:RTKN
IB:Ubiquitin
RTKN
Input
PSMD1
GAPDH

Figure 5

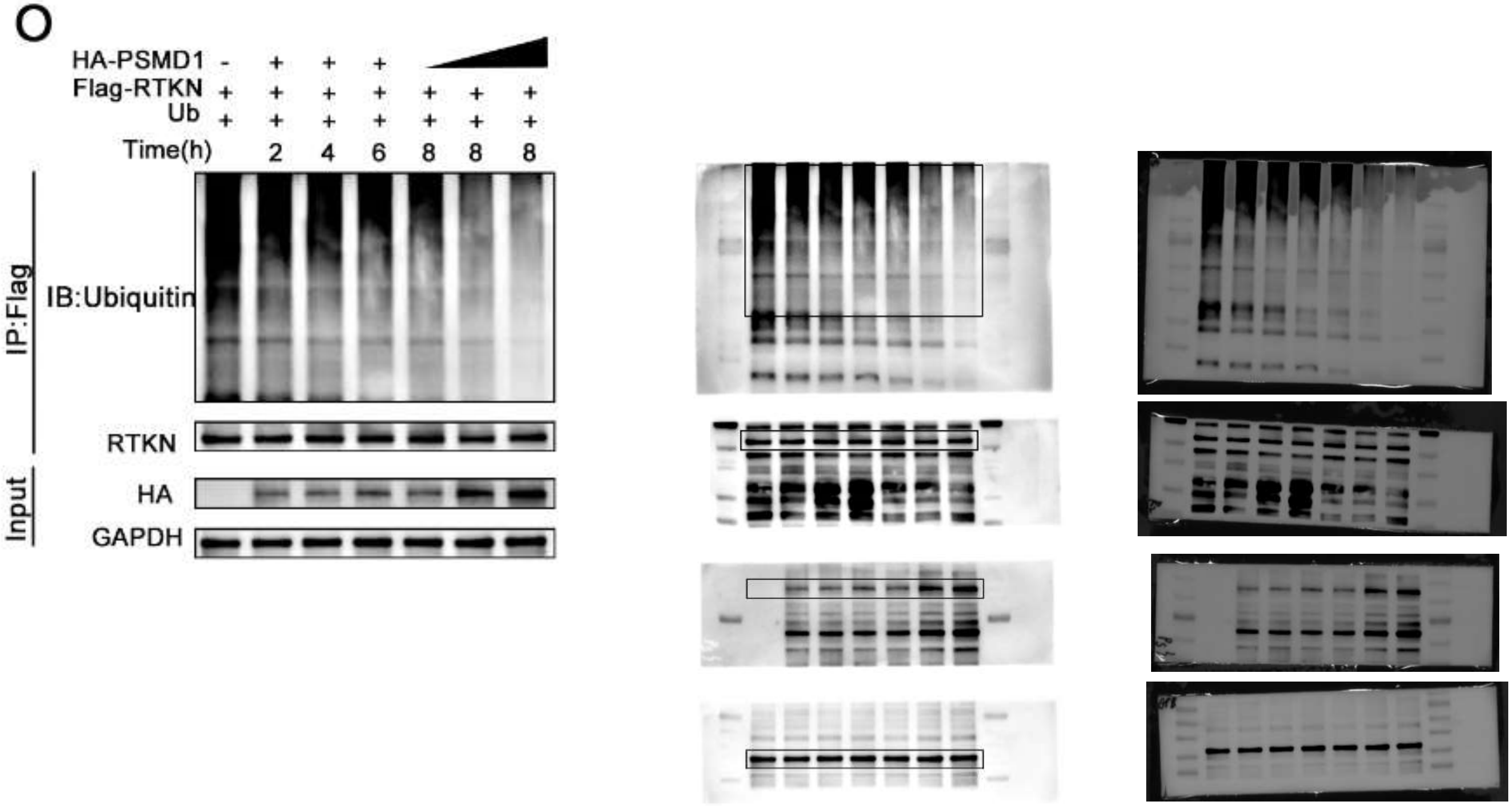
O
HA-PSMD1 - + + +
Flag-RTKN + + + + + + +
Ub + + + + + + +
Time(h) 2 4 6 8 8 8
IP:Flag
IB:Ubiquitin
RTKN
Input
HA
GAPDH

Figure 5

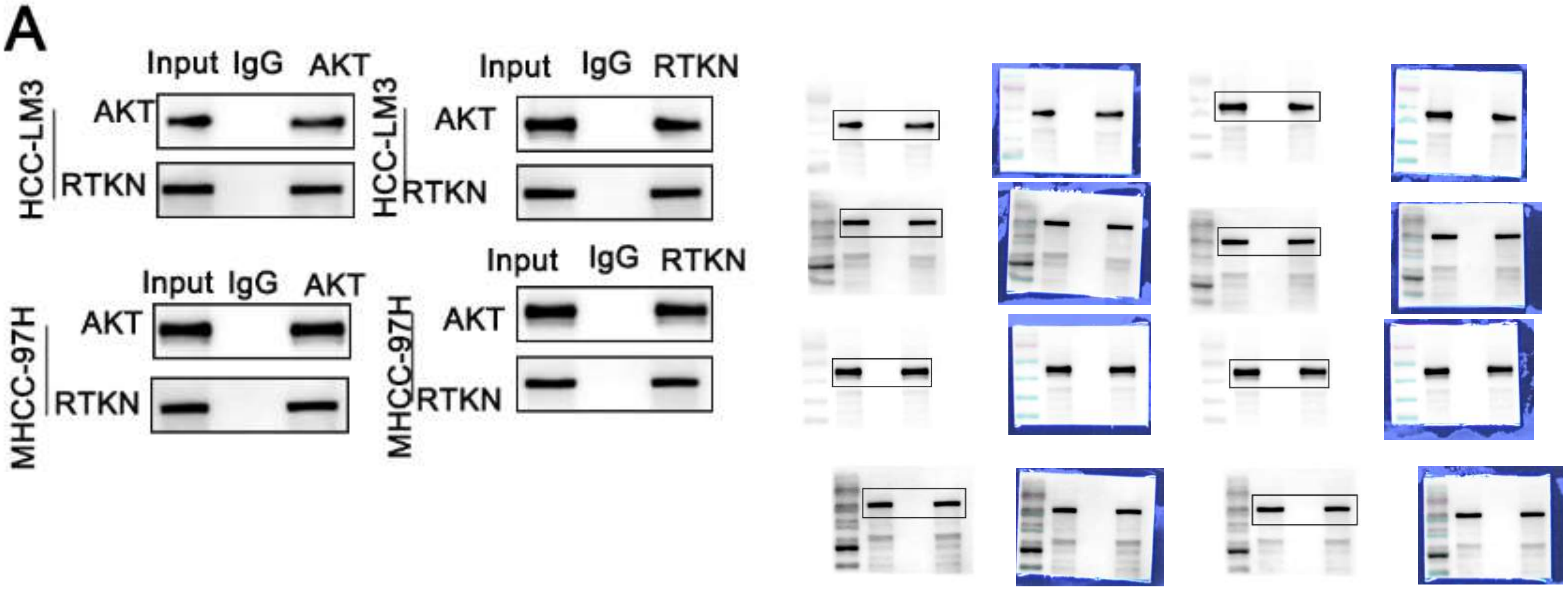
A
Input
IgG
AKT
HCC-LM3
AKT
RTKN
Input
IgG
RTKN
HCC-LM3
AKT
RTKN
Input
IgG
AKT
MHCC-97H
AKT
RTKN
Input
IgG
RTKN
MHCC-97H
AKT
RTKN

Figure 6

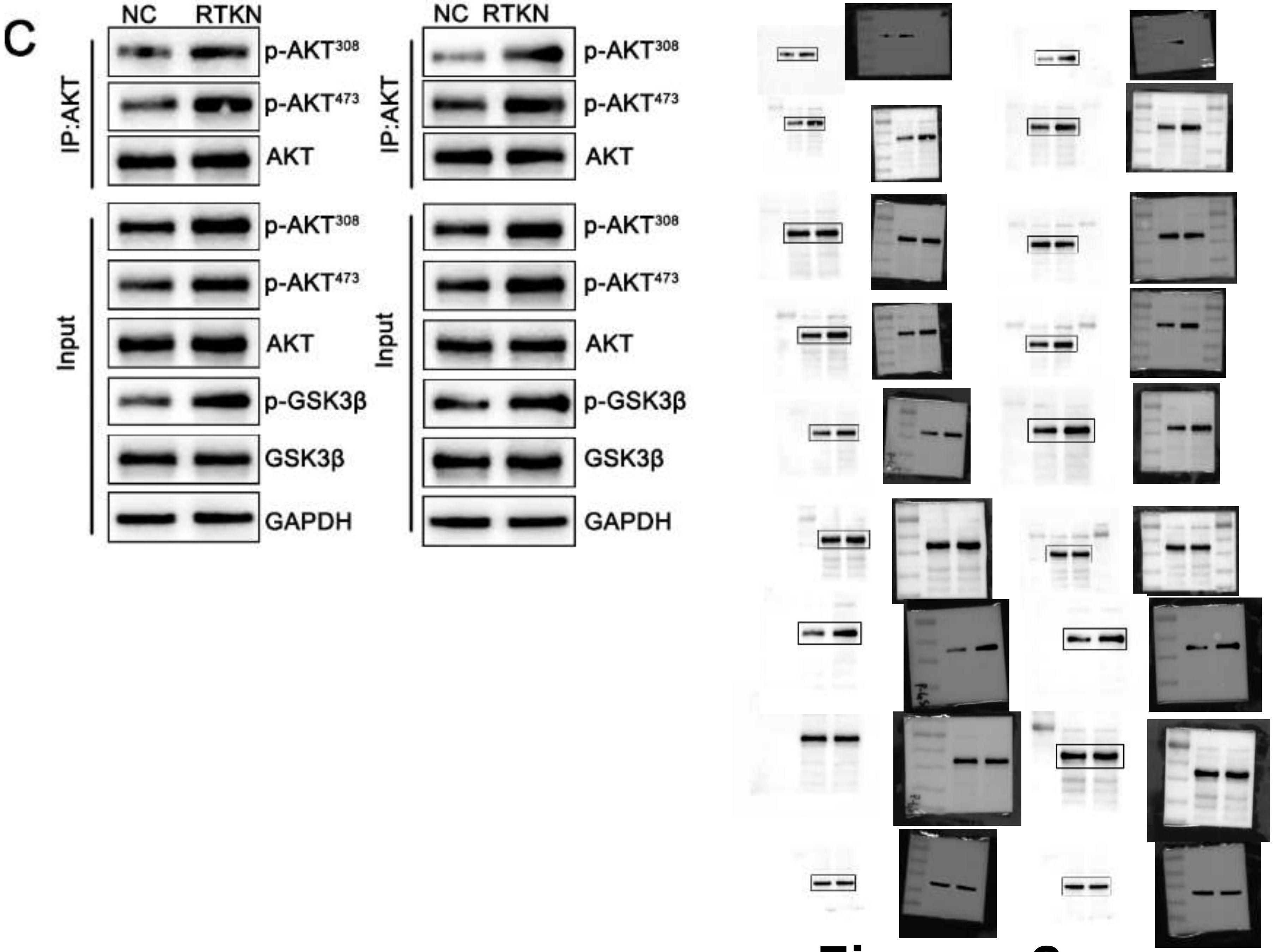
C
NC
RTKN
IP:AKT
Input
p-AKT308
p-AKT473
AKT
p-GSK3β
GSK3β
GAPDH

Figure 6

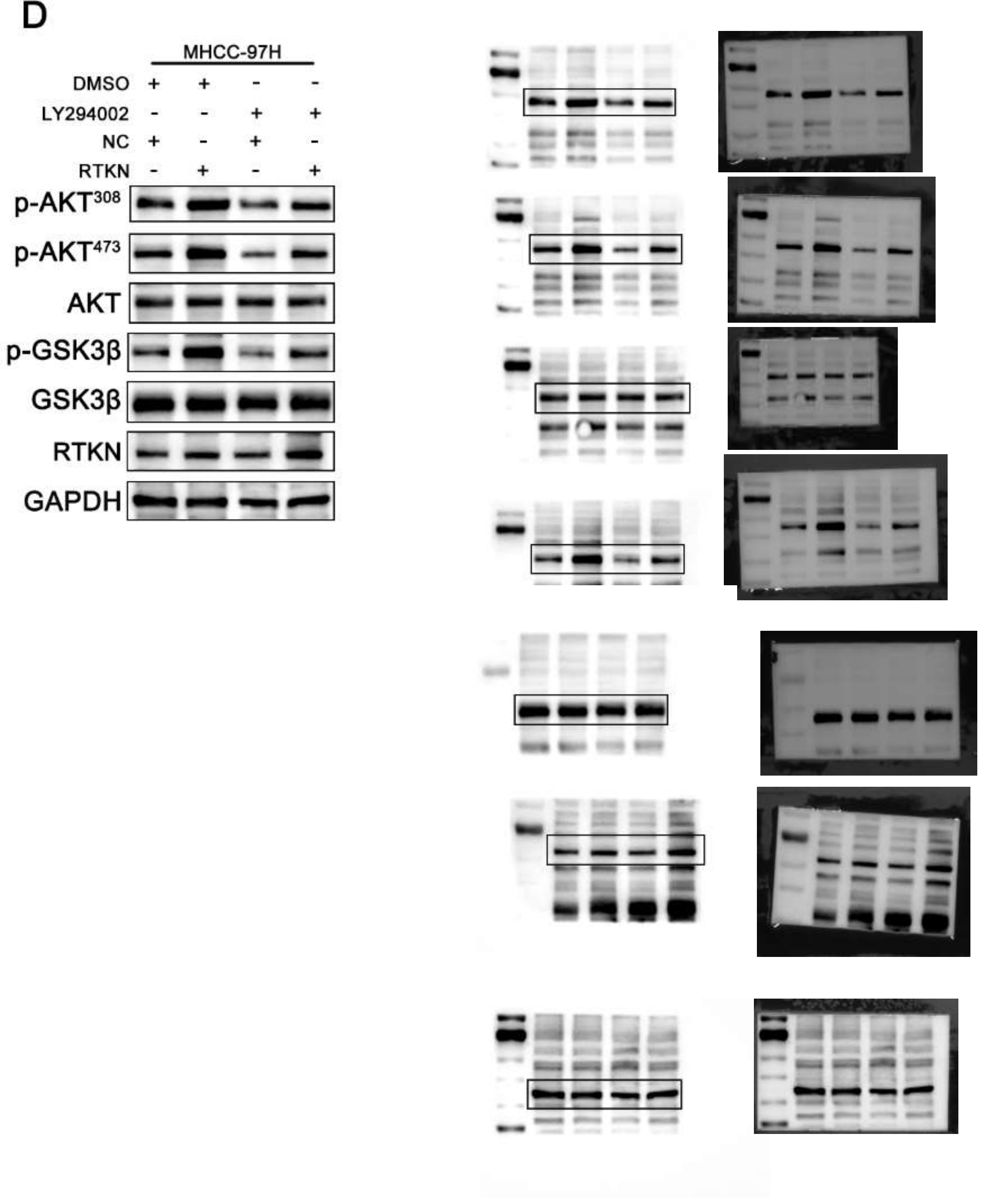
D
MHCC-97H
DMSO + + - -
LY294002 - - + +
NC + - + -
RTKN - + - +
p-AKT308
p-AKT473
AKT
p-GSK3β
GSK3β
RTKN
GAPDH

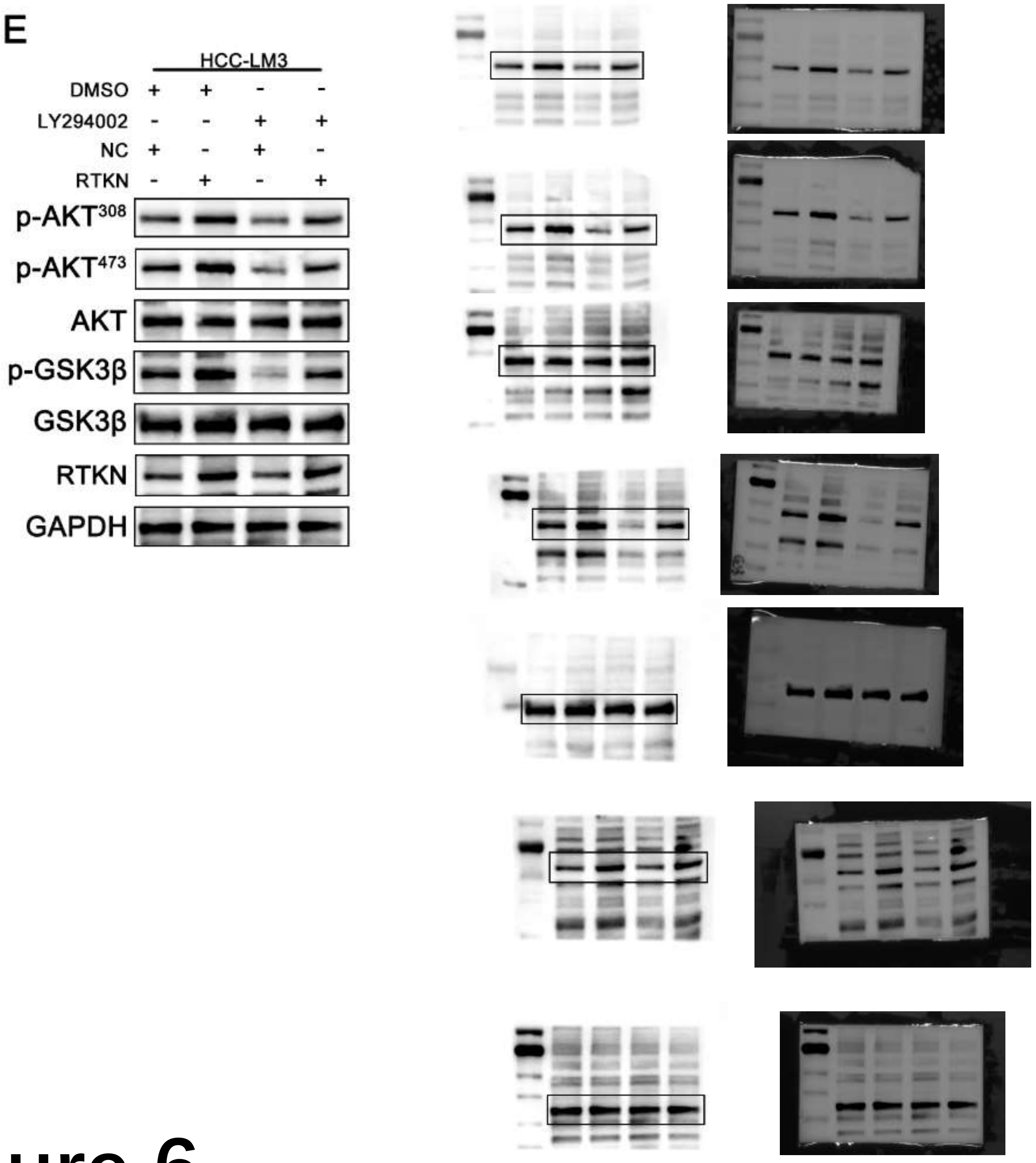
E
HCC-LM3
DMSO + + - -
LY294002 - - + +
NC + - + -
RTKN - + - +
p-AKT308
p-AKT473
AKT
p-GSK3β
GSK3β
RTKN
GAPDH

Figure 6

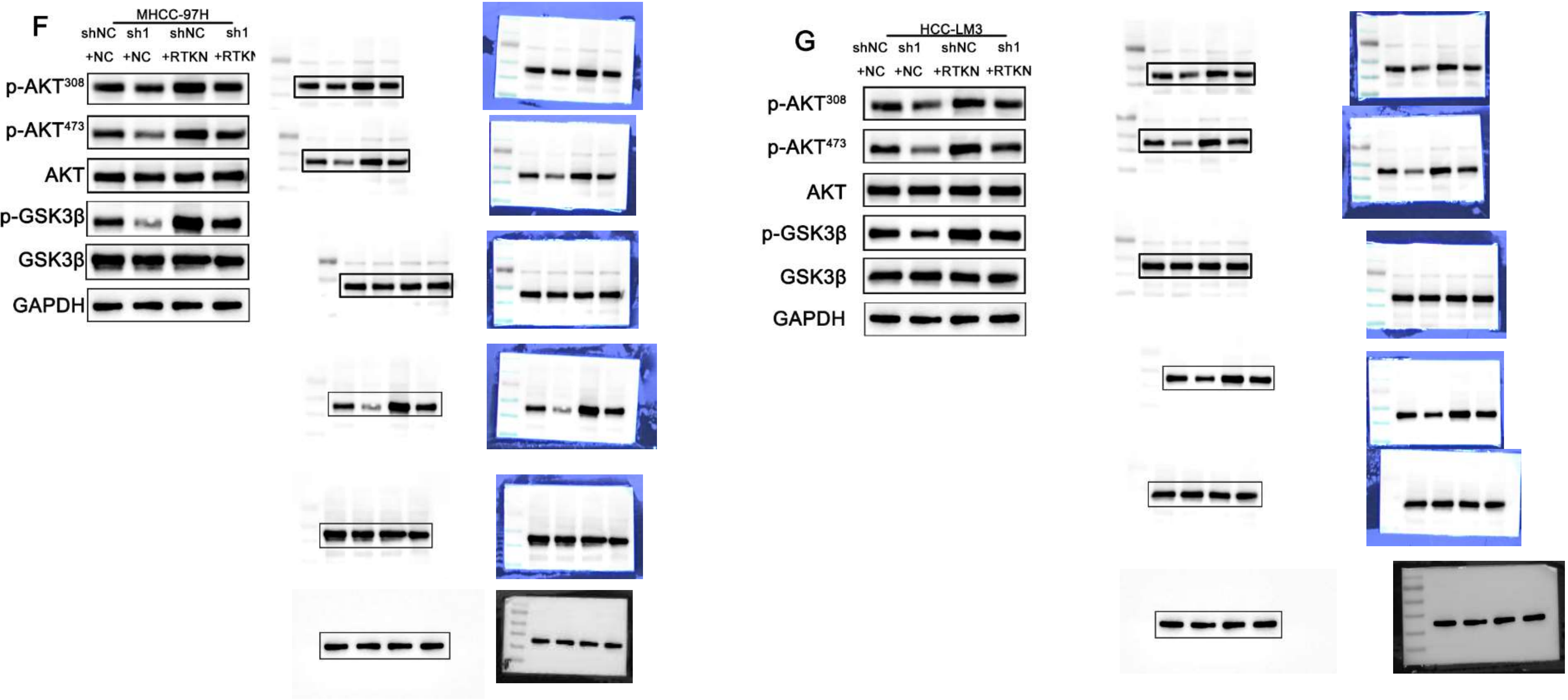
F
MHCC-97H
shNC +NC
sh1 +NC
shNC +RTKN
sh1 +RTKN
p-AKT308
p-AKT473
AKT
p-GSK3β
GSK3β
GAPDH
G
HCC-LM3
shNC +NC
sh1 +NC
shNC +RTKN
sh1 +RTKN
p-AKT308
p-AKT473
AKT
p-GSK3β
GSK3β
GAPDH

Figure 6

K

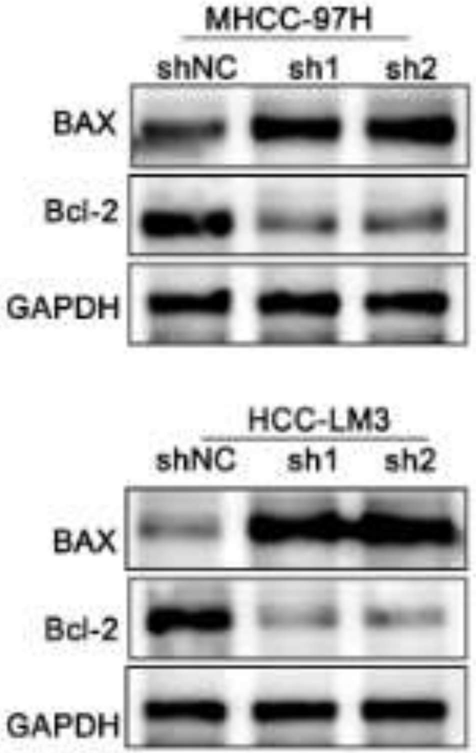
MHCC-97H
shNC
sh1
sh2
BAX
Bcl-2
GAPDH
HCC-LM3
shNC
sh1
sh2
BAX
Bcl-2
GAPDH

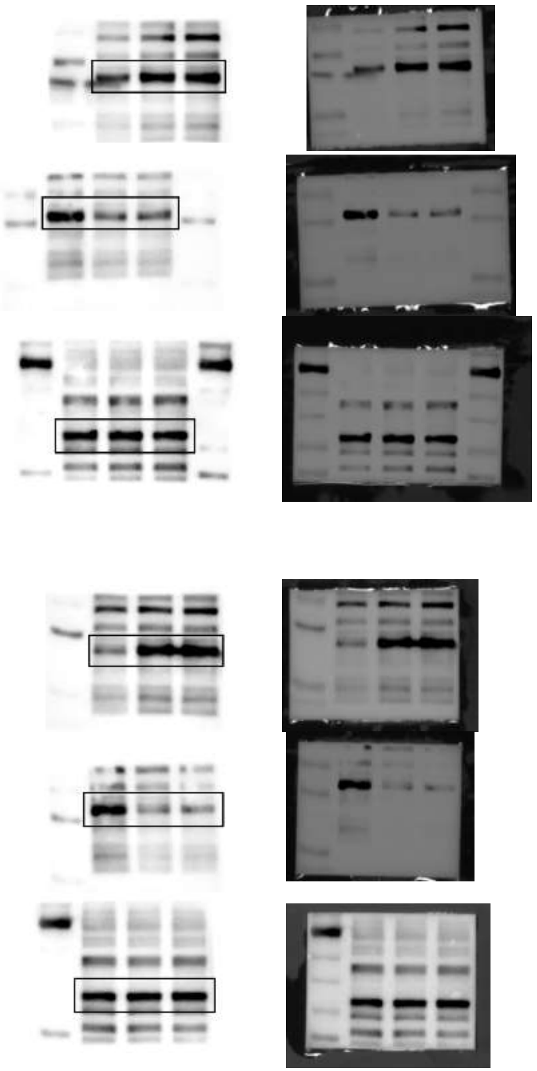

Figure S1

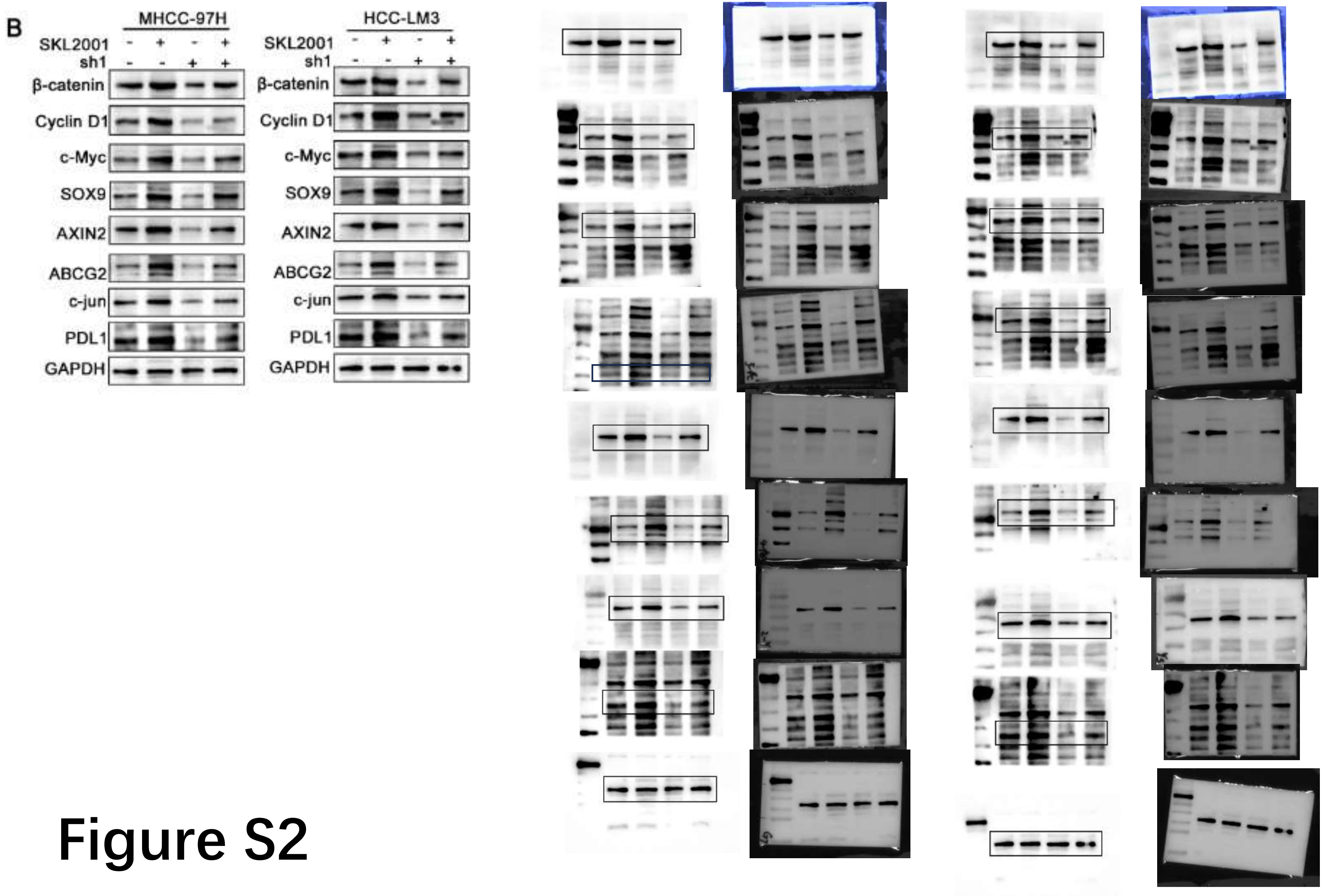
B
MHCC-97H
HCC-LM3
SKL2001 - + - +
sh1 - - + +
β-catenin
Cyclin D1
c-Myc
SOX9
AXIN2
ABCG2
c-jun
PDL1
GAPDH

Figure S2

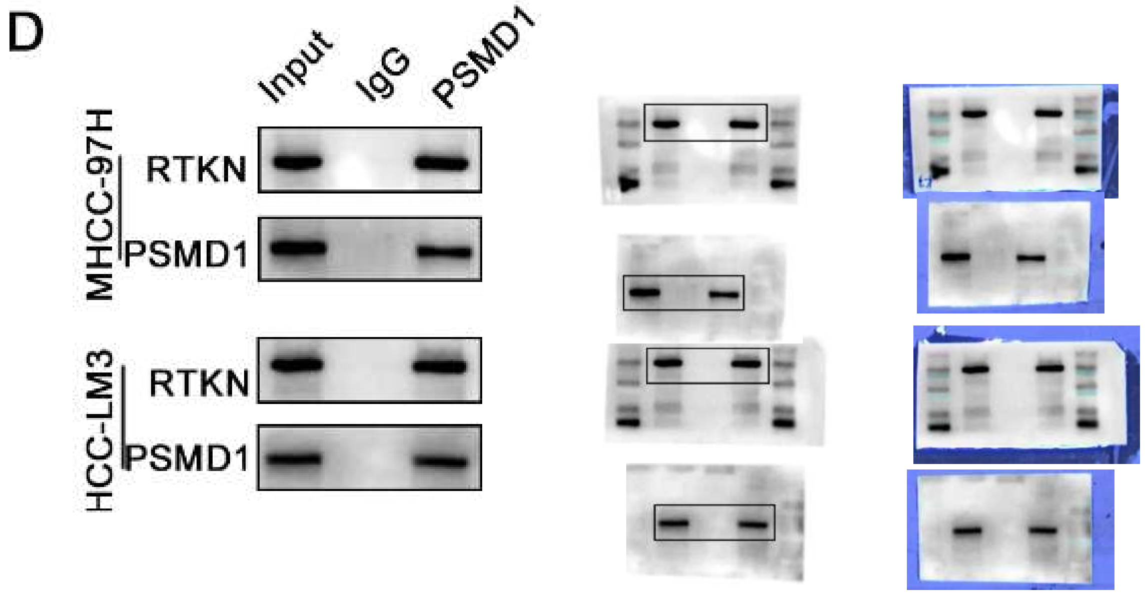
D
Input
IgG
PSMD1
MHCC-97H
RTKN
PSMD1
HCC-LM3
RTKN
PSMD1

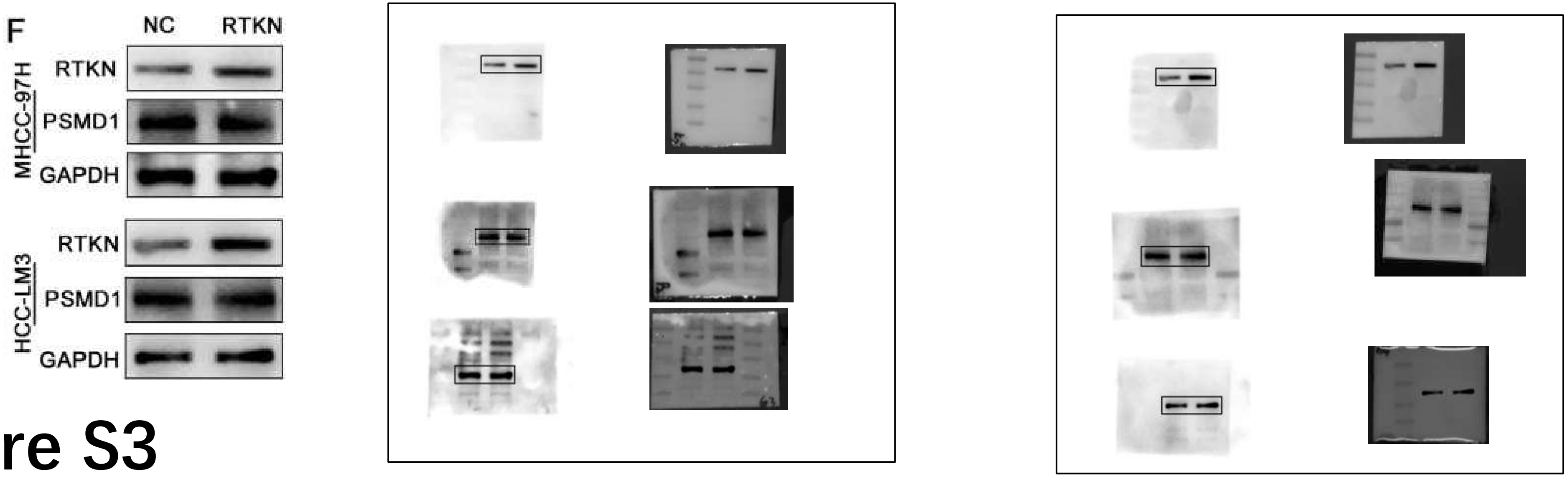
F
NC
RTKN
MHCC-97H
RTKN
PSMD1
GAPDH
HCC-LM3
RTKN
PSMD1
GAPDH

Figure S3

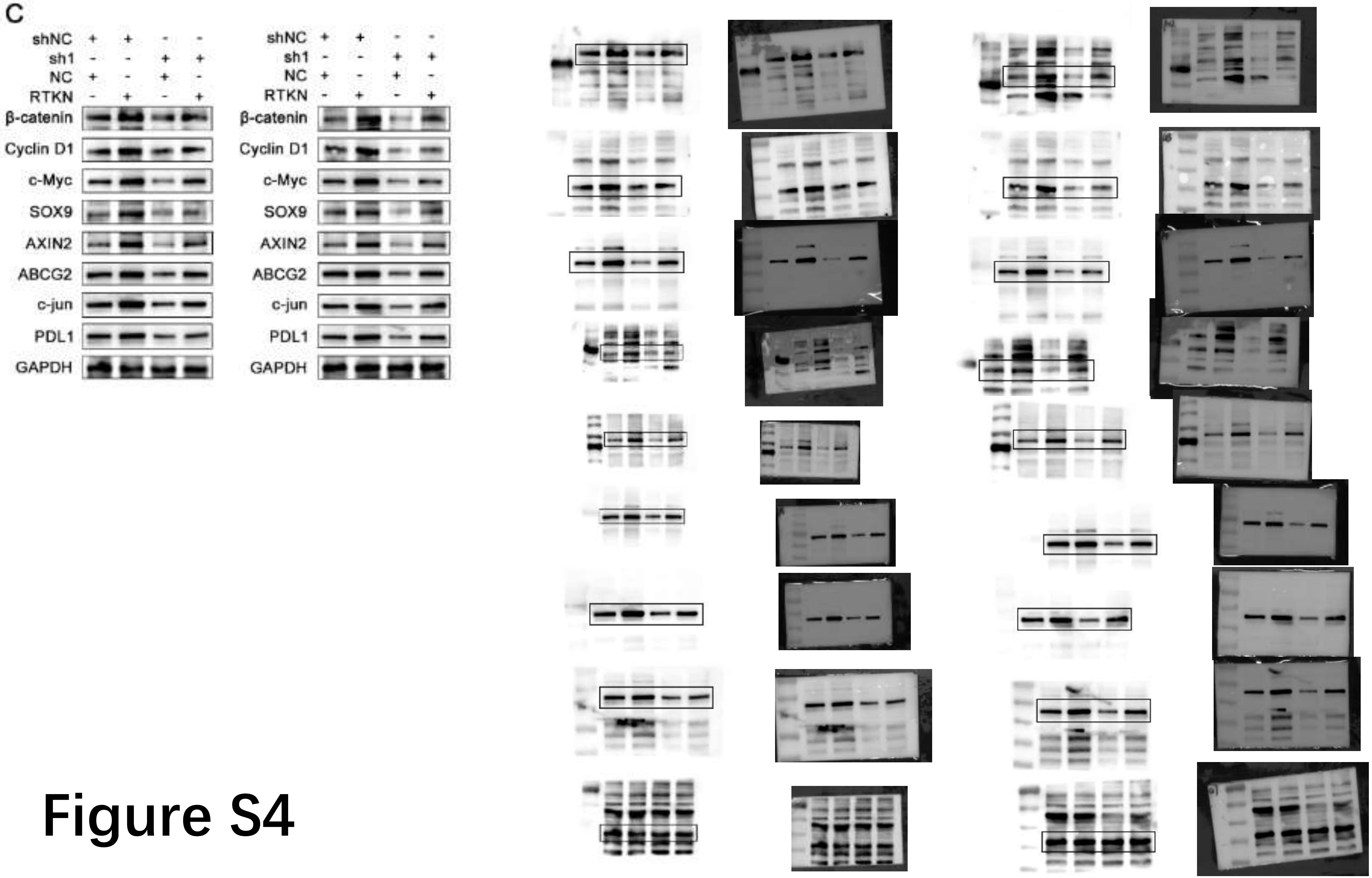
C
shNC + + - -
sh1 - - + +
NC + - + -
RTKN - + - +
β-catenin
Cyclin D1
c-Myc
SOX9
AXIN2
ABCG2
c-jun
PDL1
GAPDH

Figure S4
