# Supplementary figures and images for: PSMD1 inhibition suppresses tumor progression and enhances antitumor immunity by modulating the RTKN/β-catenin/PD-L1 axis in hepatocellular carcinoma
Source: Cell Death Dis. 2026 Jan 14;17(1):36. doi: 10.1038/s41419-025-08241-4 (PMC12804919; doi:10.1038/s41419-025-08241-4)

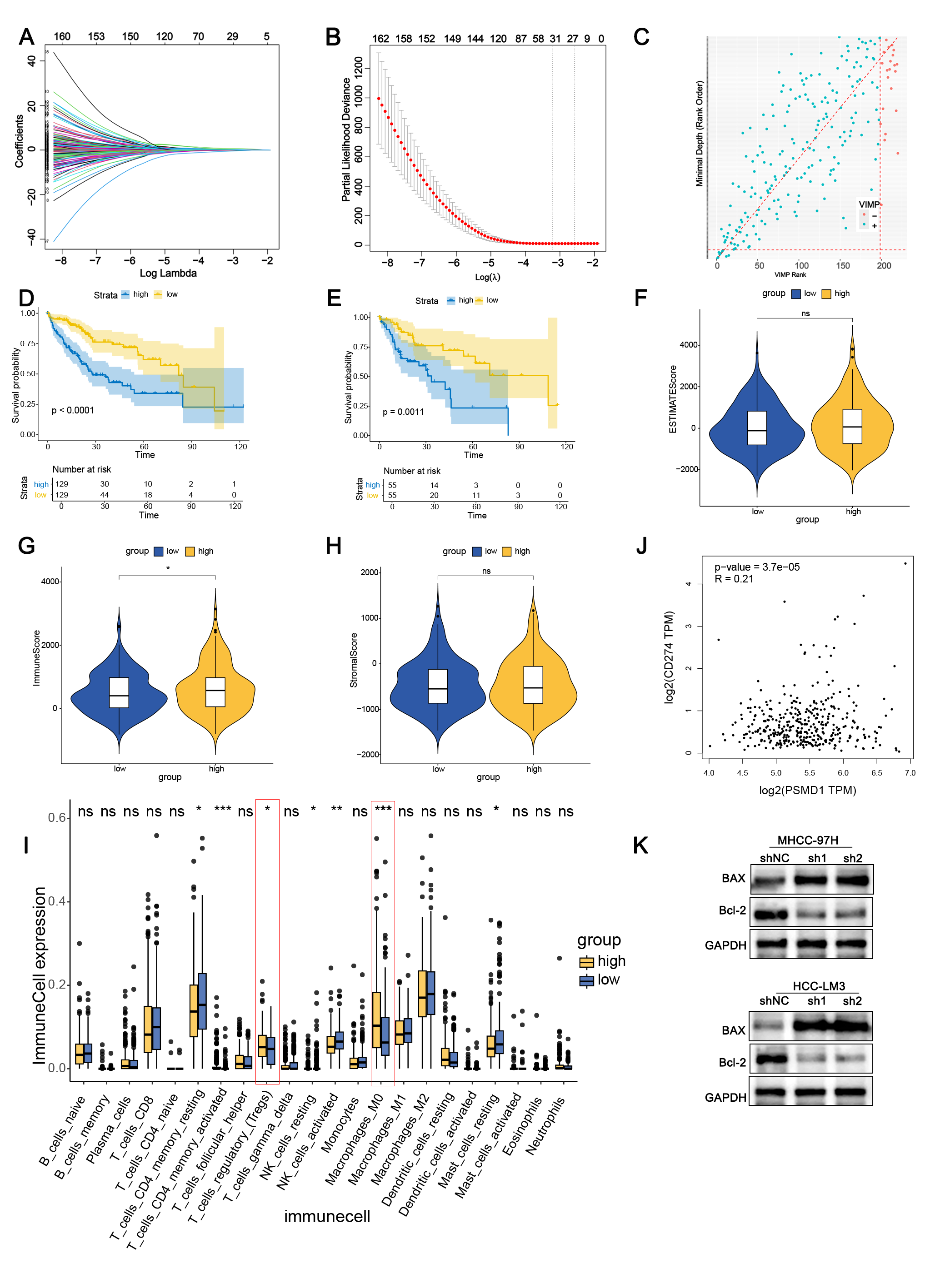

Supplement: Supplementary file 4 — Figure S1 [file 41419_2025_8241_MOESM4_ESM.tif]

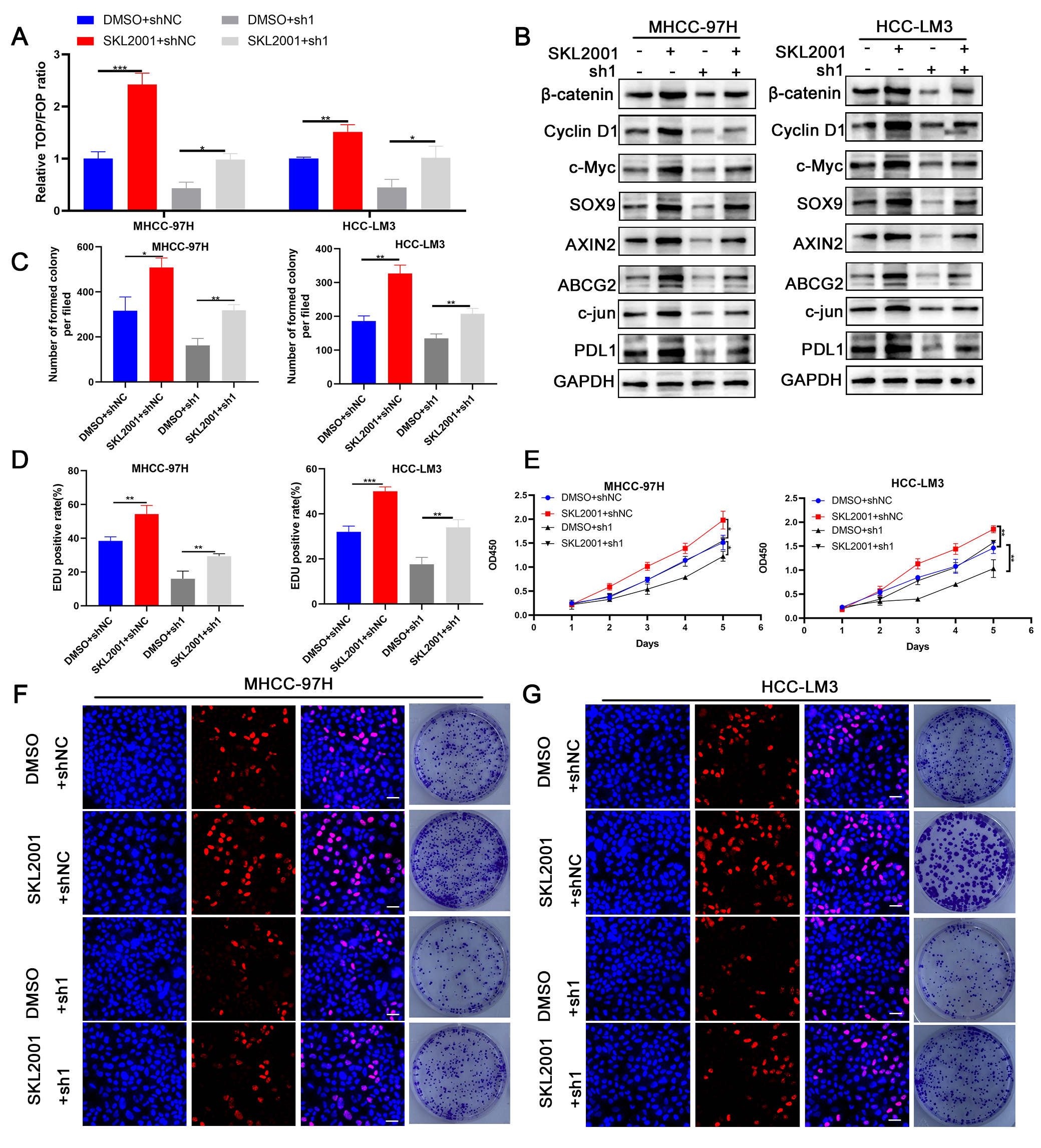

Supplement: Supplementary file 5 — Figure S2 [file 41419_2025_8241_MOESM5_ESM.tif]

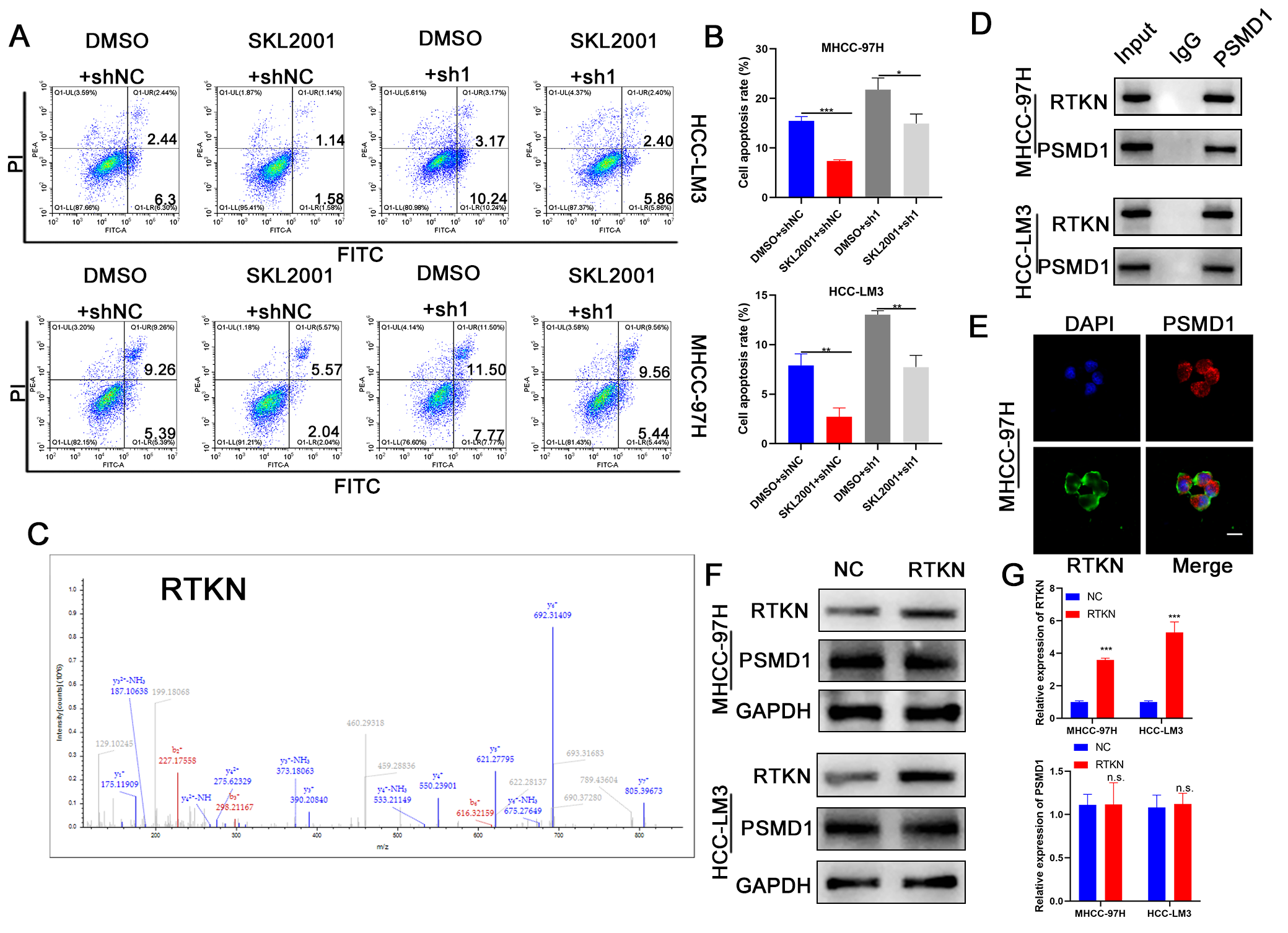

Supplement: Supplementary file 6 — Figure S3 [file 41419_2025_8241_MOESM6_ESM.tif]

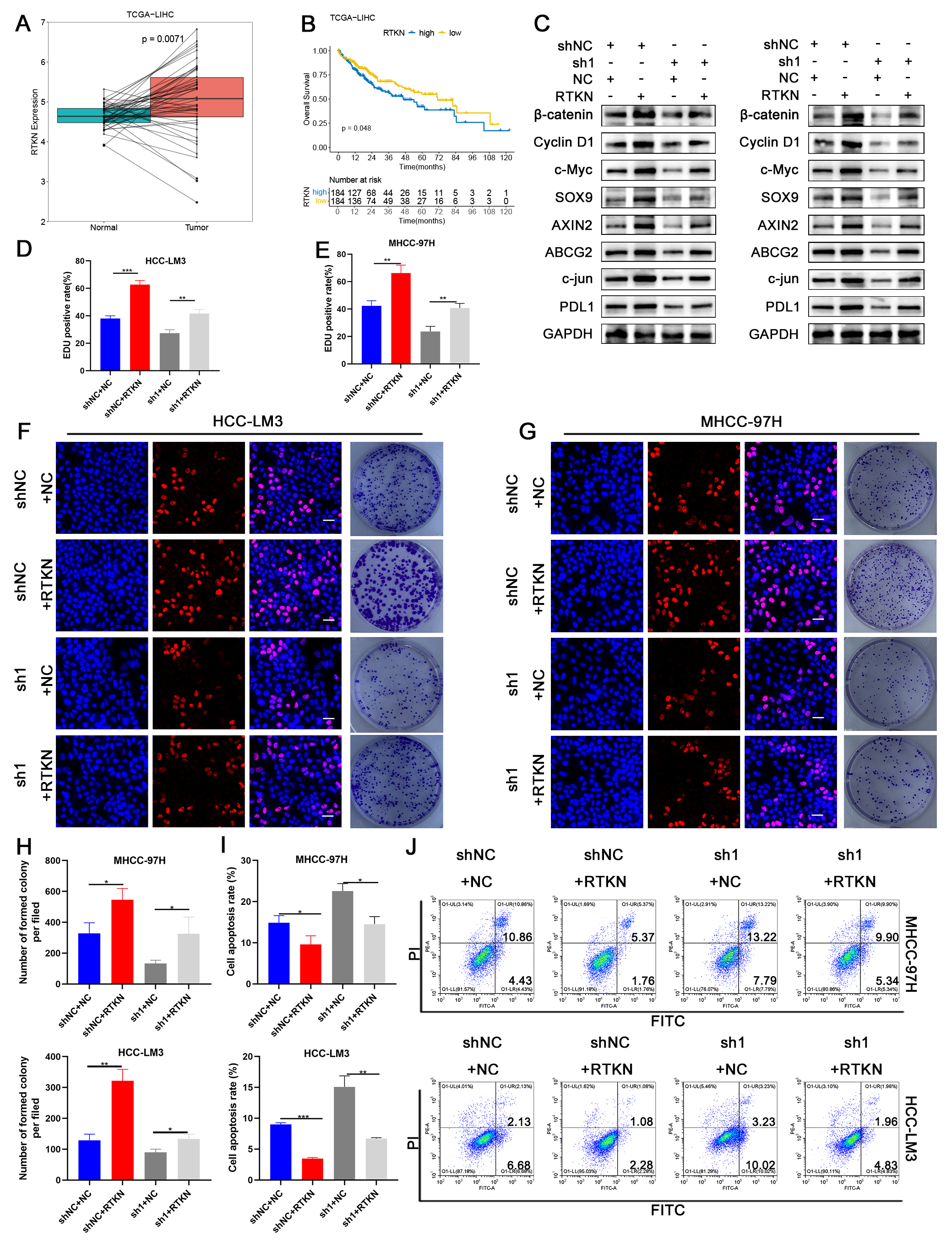

Supplement: Supplementary file 7 — Figure S4 [file 41419_2025_8241_MOESM7_ESM.tif]

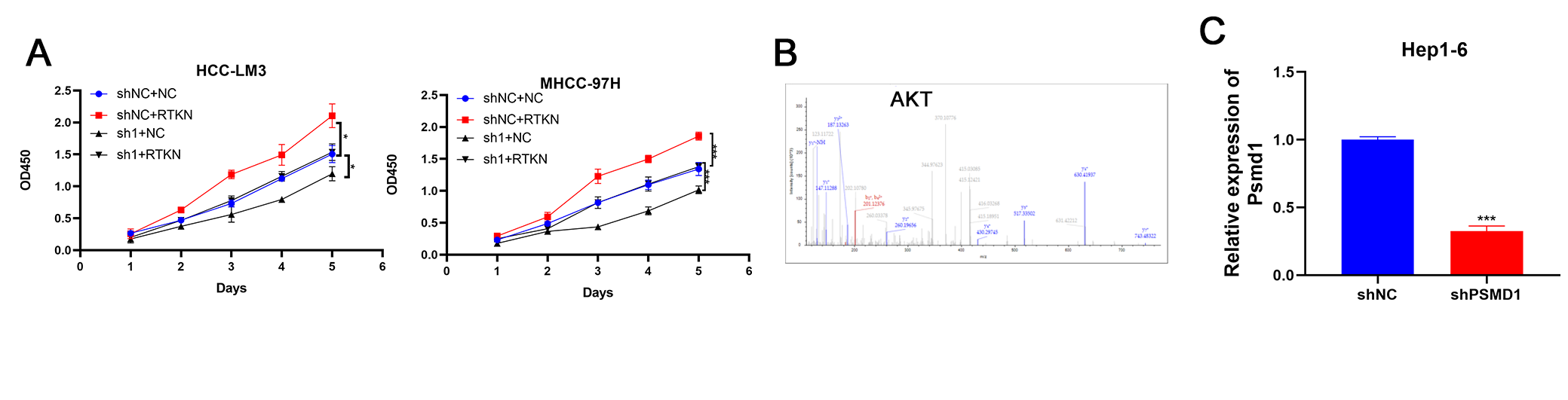

Supplement: Supplementary file 8 — Figure S5 [file 41419_2025_8241_MOESM8_ESM.tif]
