# Supplementary material for: PSMD1 inhibition suppresses tumor progression and enhances antitumor immunity by modulating the RTKN/β-catenin/PD-L1 axis in hepatocellular carcinoma
Source: Cell Death Dis. 2026 Jan 14;17(1):36. doi: 10.1038/s41419-025-08241-4 (PMC12804919; doi:10.1038/s41419-025-08241-4)
Supplement: Supplementary file 11 — Table S3 [file 41419_2025_8241_MOESM11_ESM.docx]

| PSMD1 | F: 5’-GATCCAGGCACAGAAGCAAT-3’ |
| --- | --- |
|  | R: 5’-AGGAGGACAACCTGCTGATG-3’ |
| GAPDH | F: 5’-GGAGCGAGATCCCTCCAAAAT-3’ |
|  | R: 5’-GGCTGTTGTCATACTTCTCATGG-3’ |
| CyclinD1 | F: 5′-CAGAGGCGGAGGAGAACAAA-3′ |
|  | R: 5′-ATGGAGGGCGGATTGGAA-3′ |
| c-myc | F: 5'-AAAGGCCCCCAAGGTAGTTA-3' |
|  | R: 5'-TTTCCGCAACAAGTCCTCTT-3' |
| SOX9 | F: 5'-CGACTACGCTGACCATCAGA-3' |
|  | R: 5'-AGACTGGTTGTTCCCAGTGC-3' |
| Axin2 | F: 5'-TAACCCCTCAGAGCGATGGA-3' |
|  | R: 5'-AGTTCCTCTCAGCAATCGGC-3' |
| ABCG2 | F: 5'-TCATCAGCCTCGATATTCCATCT -3' |
|  | R: 5'-GGCCCGTGGAACATAAGTCTT-3' |
| c-jun | F: 5'-ACCGACGAGCAGGAGGGCTT-3' |
|  | R: 5'-CAGCGCACCCGGGTTGAAGT-3' |
| CD274 | F:5′-GCCGACTACAAGCGAATTAC-3′ |
|  | R:5′-TCTCAGTGTGCTGGTCACAT-3′; |
| RTKN | F:5′-GCCGCTGCTTACTATTGC -3′ |
|  | R:5′-GTGCTTCCCGACTTTCTG-3′ |
| Psmd1 | F:5′-AAGACACCAGCGAAGACGT -3′ |
|  | R:5′-GTCTCAGCATGGGTTCCCTC-3′ |

**Table. S3.** The sequences of primers

Primers
