# Supplementary material for: PSMD1 inhibition suppresses tumor progression and enhances antitumor immunity by modulating the RTKN/β-catenin/PD-L1 axis in hepatocellular carcinoma
Source: Cell Death Dis. 2026 Jan 14;17(1):36. doi: 10.1038/s41419-025-08241-4 (PMC12804919; doi:10.1038/s41419-025-08241-4)
Supplement: Supplementary file 12 — Table S4 [file 41419_2025_8241_MOESM12_ESM.docx]

| **REAGENT or RESOTRCE** | **SOTRCE** | **IDENTIFIER** |
| --- | --- | --- |
| **Antibodies** | | |
| Mouse anti PSMD1 | Santacruz | sc-514808 |
| Rabbit anti-PD-L1 | CST | [13684T](https://www.cellsignal.cn/products/primary-antibodies/pd-l1-e1l3n-xp-rabbit-mab/13684) |
| Rabbit anti-β-catenin | CST | 9562S |
| Rabbit anti-Histone H3 | CST | 4499T |
| Rabbit anti-CyclinD1 | CST | 2922S |
| Rabbit anti-c-myc | CST | 5605T |
| Rabbit anti-SOX9 | CST | 82630T |
| Rabbit anti-AXIN2 | CST | 2151T |
| Rabbit anti-ABCG2 | CST | 4477S |
| Rabbit anti-C-JUN | CST | 9165T |
| Rabbit anti-HA | CST | 3724T |
| Rabbit anti-RTKN | Proteintech | 12762-1-AP |
| Rabbit anti-p-AKT^308^ | CST | 9275S |
| Rabbit anti-p- AKT ^473^ | CST | 4060T |
| Rabbit anti-AKT | CST | 9272S |
| Rabbit anti-p-GSK3β | CST | 5558T |
| Rabbit anti-GSK3β | CST | 9315T |
| Rabbit anti-CD8a | CST | 98941T |
| Rabbit anti-Bcl2 | CST | 4223T |
| Rabbit anti-Bax | CST | 2772T |
| Rabbit anti-Flag | CST | 14793T |
| Rabbit anti-Ki67 | CST | 9129T |
| Rabbit Anti- GADPH | CST | 5174T |
| Rabbit anti-ubiquitin | Proteintech | 10201-2-AP |
| Anti-rabbit IgG, HRP-linked Antibody | CST | 7074S |
| Alexa Fluor 594-conjugated goat anti-Rabbit IgG | Invitrogen | A-11012 |
| Alexa Fluor 488-conjugated goat anti-Rabbit IgG | Invitrogen | A-11008 |
| Anti-IgG | Abcam | ab172730 |
|  |  |  |
|  |  |  |
|  |  |  |
|  |  |  |
|  |  |  |

**Table. S4. Antibodies**
