# Supplementary material for: PSMD1 inhibition suppresses tumor progression and enhances antitumor immunity by modulating the RTKN/β-catenin/PD-L1 axis in hepatocellular carcinoma
Source: Cell Death Dis. 2026 Jan 14;17(1):36. doi: 10.1038/s41419-025-08241-4 (PMC12804919; doi:10.1038/s41419-025-08241-4)
Supplement: Supplementary file 13 — Table S5 [file 41419_2025_8241_MOESM13_ESM.docx]

| h-ShPSMD1-1 | GCACGTCAAGATGTTTATGAT |
| --- | --- |
| h-ShPSMD1-2 | GCTGTAAGTGATGTTAATGAT |
| h-ShPSMD1-3 | GCATCATCATTCTGAAGGATA |
| m-shPsmd1 | GGATAATCCAGCACGAGTTAT |
|  |  |
|  |  |

**Table s5. shRNAs sequences**
